# Surface-ligand-induced crystallographic disorder–order transition in oriented attachment for the tuneable assembly of mesocrystals

**DOI:** 10.1038/s41467-022-28830-7

**Published:** 2022-03-03

**Authors:** Bum Chul Park, Min Jun Ko, Young Kwang Kim, Gyu Won Kim, Myeong Soo Kim, Thomas Myeongseok Koo, Hong En Fu, Young Keun Kim

**Affiliations:** 1 Department of Materials Science and Engineering, Korea University, Seoul, 02841 Republic of Korea; 2 Brain Korea Center for Smart Materials and Devices, Korea University, Seoul, 02841 Republic of Korea; 3 Virtual Lab Inc, Seoul, 04799 Republic of Korea; 4 Institute of High Technology Materials and Devices, Korea University, Seoul, 02841 Republic of Korea

**Keywords:** Nanoparticles, Magnetic materials

## Abstract

In the crystallisation of nanomaterials, an assembly-based mechanism termed ‘oriented attachment’ (OA) has recently been recognised as an alternative mechanism of crystal growth that cannot be explained by the classical theory. However, attachment alignment during OA is not currently tuneable because its mechanism is poorly understood. Here, we identify the crystallographic disorder-order transitions in the OA of magnetite (Fe_3_O_4_) mesocrystals depending on the types of organic surface ligands on the building blocks, which produce different grain structures. We find that alignment variations induced by different surface ligands are guided by surface energy anisotropy reduction and surface deformation. Further, we determine the effects of alignment-dependent magnetic interactions between building blocks on the global magnetic properties of mesocrystals and their chains. These results revisit the driving force of OA and provide an approach for chemically controlling the crystallographic order in colloidal nanocrystalline materials directly related to grain engineering.

## Abstract (web summary)

Oriented attachment is a non-classical growth mechanism of nanomaterials that can lead to tunable properties and functionalities. Here the authors show that the crystallographic alignment between magnetite mesocrystal building-blocks can be tuned by the surface ligands, influencing the resulting magnetic properties.

## Introduction

The assembly of nanometric building blocks into self-limited periodic structures is key to forming natural and artificial materials and in their structure-induced emergent properties^1–6^. In the field of mesocrystal crystallisation, during which the building blocks regularly tile into discernible single crystals, an assembly-based growth mechanism referred to as ‘oriented attachment’ (OA) has entered into the spotlight as a new model for explaining the creation of crystallographically ordered structures with emergent functionalities that cannot be explained by the classical nucleation and growth theory, occurring via ion-by-ion accretion^7–9^. In OA, crystal growth proceeds as building-block nanocrystals (BNCs) adhere to each other in the same crystallographic order; this mechanism is widely observed in the formation of various natural and chemically synthesised nanomaterials such as metal oxides (e.g., iron oxides, TiO_2_)^10–12^, ceramics (PbSe)^13^, and even metals (Au)^14^. Accordingly, OA has advanced our understanding of the growth of complex hierarchical and anisotropic nanostructures as well as the evolution of microstructural features, including twins, stacking faults, and grain boundaries.

Therefore, discovering an unknown rule in crystallisation via OA into mesocrystals is an important goal, particularly in studying the nonclassical crystallisation of iron oxides to control their magnetic properties and tailor them for promising applications in biomedicine and electronics^15,16^. Several groups successfully proposed the thermodynamic contribution of the volume free energy of BNC formation to the phase transformation into final magnetite (Fe_3_O_4_)-phase nanoparticles^17^ and to size-predictable nanoparticle synthesis by interpreting the kinetics of the colloidal assembly of Fe_3_O_4_ BNCs^18^. Recently, another group proposed a new OA pathway for the growth of hematite (*α*-Fe_2_O_3_) via interface-driven nucleation and attachment, in which primary particles nucleate and attach at a distance of ~2 nm from the parent mesocrystal in the presence of a surface ligand, namely, oxalate^8^.

Despite these contributions, it remains unknown how the BNCs are crystallographically aligned during OA and whether the BNC alignment is chemically controllable by overcoming the confined order of OA. Concomitantly, another unanswered question is how the magnetic properties of mesocrystals vary with the crystallographic order of the BNCs. The magnetic properties of disordered and ordered nanocrystalline materials are theoretically well understood. However, experimentally elucidating this effect remains challenging because the crystallographic order of intra-mesocrystal grains has never been successfully controlled during chemical synthesis^19^.

This study demonstrates that the crystallographic alignment between BNCs with truncated octahedral shapes can be tuned during the OA of Fe_3_O_4_ mesocrystals by coordinating different surface ligands, including acetate, polyacrylate, and Mg^2+^-adsorbed polyacrylate on the BNCs. The experiments and density functional theory (DFT) calculations presented herein reveal that the tiling rule of BNCs is guided by the reduction in the surface energy anisotropy and surface deformation commensurate to the coordination of different surface ligands, which could not be predicted using generic interparticle interactions following the Derjaguin–Landau–Verwey–Overbeek (DLVO) theory^20^. BNC alignment significantly influences inter-BNC magnetic interactions, resulting in different collective magnetic behaviours of individual Fe_3_O_4_ mesocrystals and their magnetic chains.

## Results

### Crystallisation of Fe_3_O_4_ mesocrystal

**Fig. 1:**
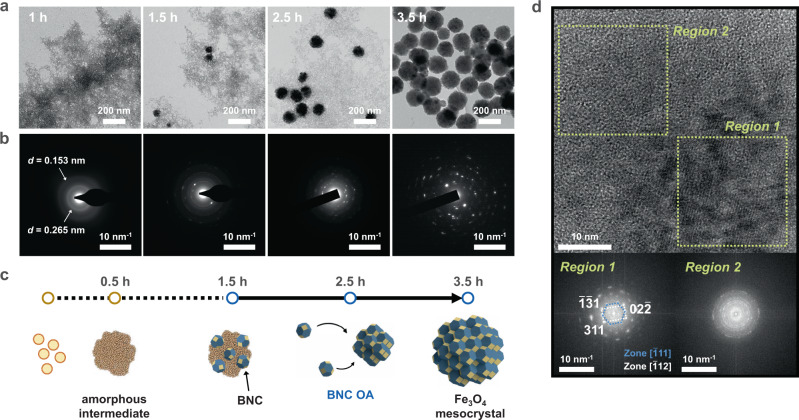
Control of Fe_3_O_4_ mesocrystal crystallisation pathway via OA. **a** TEM images and **b** SAED patterns of Fe oxyhydroxide intermediates and Fe_3_O_4_ mesocrystals at different reaction times. **c** Crystallisation pathway of Fe_3_O_4_ mesocrystals including stepwise transformation (dotted line) and OA (solid line). **d** HR-TEM image at the boundary between crystalline Fe_3_O_4_ (Region 1) and the poorly crystalline Fe oxyhydroxide intermediate phase (Region 2) and corresponding FFT images.

**Fig. 2:**
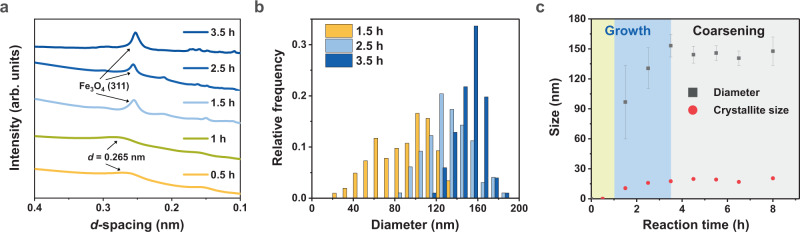
Growth of Fe_3_O_4_ mesocrystal. **a** Radial distribution profiles from the SAED patterns at all reaction stages. The position of main diffraction peaks originated from the intermediate phase is marked as *d* = 0.265 nm. **b** Size distributions obtained by measuring 200 Fe_3_O_4_ mesocrystals. **c** Kinetic approximation of Fe_3_O_4_ growth, indicating that the mesocrystal and crystallite sizes are similar after 3.5 h. Each data point was obtained from the mean of the Gaussian distribution (*n* = 200) and Debye–Scherrer equation. The error bars represent standard deviations.

We synthesised Fe_3_O_4_ mesocrystals using the modified polyol method and observed their crystallisation process via ex situ transmission electron microscopy (TEM) (Fig. 1). Specifically, we reacted a mixture of sodium acetate (hydroxyl ion source) dissolved in ethylene glycol and iron chloride hexahydrate (ferric ion source) dissolved in H_2_O for 15 min at room temperature and subsequently for 210 min at 200 °C (see Methods), yielding Fe_3_O_4_ mesocrystals. TEM observations reveal that the Fe_3_O_4_ mesocrystals did not form directly from ions but rather formed through a stepwise transformation while consuming a ferric oxyhydroxide intermediate as a precursor for Fe_3_O_4_ growth (Fig. 1a–d). At the beginning of the reaction (*t* = 0.5 h), a broad ring diffraction pattern appeared in the selected area electron diffraction (SAED) images at *d*-spacings of 0.265 and 0.153 nm, indicating the formation of poorly crystalline ferric oxyhydroxide with short-range order (Figs. 1b and 2a and Supplementary Fig. 1)^21^. We also verified that the oxidation state of Fe in this intermediate was the ferric state (Fe^3+^), through the electron energy-loss spectroscopy (EELS) of the Fe 2*p L*_3_ edge with a peak at 711.8 eV (Supplementary Fig. 1)^22^. After *t* = 1.5 h, diffraction spots corresponding to Fe_3_O_4_ gradually appeared, with the main peak attributed to the {311} plane with a *d*-spacing of 0.253 nm. By *t* = 3.5 h, the intermediate phase had been entirely consumed, leading to the growth of Fe_3_O_4_ mesocrystals with a uniform size of 153 ± 11 nm (Fig. 2b, c). The Fe_3_O_4_ mesocrystals that initially formed in the intermediate matrix had a broad size distribution, but this distribution gradually narrowed with increasing reaction time (Fig. 2b). In addition, the average Fe_3_O_4_ mesocrystal diameter increased with increasing initial [Fe^3+^] content, in agreement with growth via OA (Supplementary Fig. 2)^18^.

The polymorphism of ferric oxyhydroxide as an intermediate significantly affects the crystallisation mechanism of Fe_3_O_4_ mesocrystals^17,18,23–25^. We recently identified two Fe_3_O_4_ crystallisation pathways starting from either lepidocrocite or goethite and revealed that Fe_3_O_4_ mesocrystals grew via OA only in the former case^23^. These pathways are competitively responsible for Fe_3_O_4_ growth and can be selectively regulated according to the initial [OH^–^]/[Fe^3+^] ratio. In this study, we chemically controlled Fe_3_O_4_ mesocrystals such that they grow through a single crystallisation pathway via OA (Supplementary Figs. 3–5 and Supplementary Note 1).

As shown in the high-resolution (HR) TEM image (Fig. 1d), at *t* = 1.5 h, the fast Fourier transform (FFT) pattern of a nanogranular Fe_3_O_4_ mesocrystal comprising assemblies of smaller BNCs (Region 1) exhibits a spot diffraction pattern, indicating that the BNCs have almost identical orientations. In contrast, the polycrystalline ring pattern at the periphery of the nanogranular mesocrystal (Region 2) is attributed to the random orientation of Fe_3_O_4_ BNCs formed in the lepidocrocite intermediate. This secondary structure, in which the primary particles are assembled in a specific crystallographic order in the range from hundreds of nanometres to micrometres, is characteristic of mesocrystals. These mesocrystalline structures typically form through the colloidal assembly of BNCs.

### Tuneable crystallographic order in OA

**Fig. 3:**
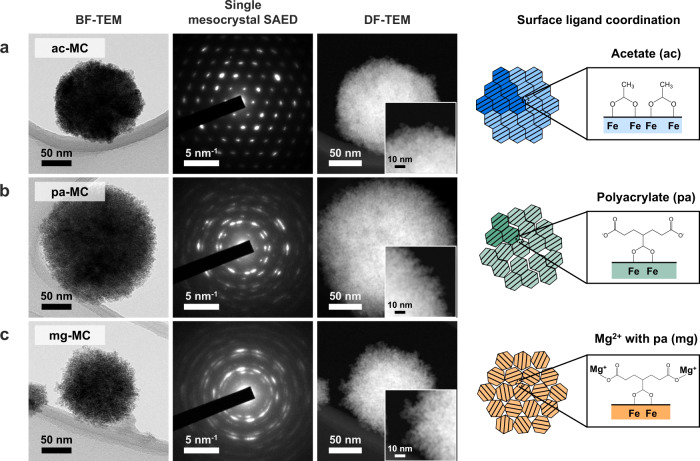
Disorder–order transition in the crystallographic alignment in mesocrystals during OA. **a**–**c** Morphological and microstructural analyses of Fe_3_O_4_ mesocrystals with different crystallographic orders in BNC tiling depending on the surface ligand: acetate (ac) (**a**), polyacrylate (pa) (**b**), and Mg^2+^-adsorbed polyacrylate (mg) (**c**). Bright-field (BF)-TEM images display a representative shape for each mesocrystal, and the SAED pattern was acquired from the single mesocrystal in the TEM images. Dark-field (DF)-STEM images describe the change in the outline of single mesocrystals.

We examined the morphologies and crystallographic orientations of individual single mesocrystals coordinated with different surface ligands (Fig. 3), which included acetate (denoted in sample labels as ‘ac’), polyacrylate (‘pa’), and Mg^2+^-adsorbed polyacrylate (‘mg’) as model moieties that can coordinate on the Fe_3_O_4_ BNC surface as a monolayer during the reaction (see Methods).

Through a surface analysis using Fourier transform infrared (FT-IR) spectroscopy, X-ray photoelectron spectroscopy (XPS), and thermogravimetric–differential thermal analysis (TG-DTA), we confirmed that the ligand densities for polyacrylate (1.92 ligand nm^−2^) and Mg^2+^-adsorbed polyacrylate (2.57 ligand nm^−2^) on BNCs were close to the maximum ligand-to-surface ratio for the coordinated monolayer (2.4–2.7 ligand nm^−2^), whereas that for the acetate-coordinated surface (2.42 ligand nm^−2^) was much lower than the theoretical maximum value (9 ligands nm^−2^) required to sufficiently fill the BNC surface (Supplementary Fig. 6 and Supplementary Table 1)^26^. In the FT-IR results, the average shifts of Δ(*ν*_*as*_(COOFe)–*ν*_*s*_(COOFe)) for the acetate-coordinated mesocrystals (ac-MCs) and polyacrylate-coordinated mesocrystals (pa-MCs) were both 170 cm^−1^, similar to that for pure deprotonated polyacrylate. Furthermore, no C=O vibration peak appeared at ~1700 cm^−1^, indicating that the carboxyl groups of ligands were coordinated to Fe on the BNCs via bidentate bridging (Supplementary Fig. 7 and Supplementary Table 2)^26,27^. Both the BNC surfaces of pa-MC and Mg^2+^-adsorbed polyacrylate-coordinated mesocrystals (mg-MCs) had free deprotonated carboxyl acid groups that were not bonded to Fe, as manifested by the FT-IR peaks at 1410 cm^−1^ and the increasing intensity of the C 1*s* and O 1*s* XPS peaks (Supplementary Fig. 6 and Supplementary Table 3). The methyl group of the acetate ligand reduced the zeta potential to nearly 0 mV, and the unbound free carboxylates of polyacrylate led to a negatively charged surface with a zeta potential of −25 mV (Supplementary Fig. 7). The negative charge of polyacrylate can attract Mg^2+^ near the BNC surface through electrostatic interactions; which is responsible for the presence of 5 at% of Mg compared to Fe and the increasing intensity of the Mg 1*s* XPS peak (Supplementary Figs. 6 and 8)^28^.

**Fig. 4:**
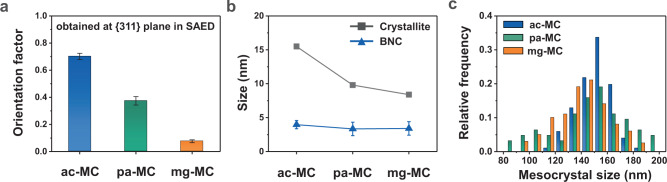
Quantitative analyses of crystallographic alignment. **a** Orientation factor obtained from the azimuthal profile of the SAED pattern of a single mesocrystal at a constant distance corresponding to the {311} plane. **b** Crystallite sizes calculated using the Debye–Scherrer equation and measured from the HR-TEM images. **c** Size distribution of each sample measured from 50 mesocrystals in the TEM images. Error bars in panels (**a**) and (**b**) indicate standard deviations.

All the Fe_3_O_4_ mesocrystals were uniformly synthesised with similar sizes: 153 ± 11 nm for the ac-MCs, 153 ± 16 nm for the pa-MCs, and 141 ± 20 nm for the mg-MCs (Fig. 4c). Surprisingly, the BNC alignment changed depending on the surface ligands. For ac-MC, BNCs were attached in the same crystallographic direction, resulting in a single-crystal-like spot diffraction pattern (Fig. 3a). When polyacrylate was grafted onto the BNC surface, the diffraction spots were stretched owing to the slight misalignment between BNCs (Fig. 3b). Furthermore, when Mg^2+^ was attracted to the negatively charged polyacrylate, the BNCs did not exhibit regular alignment, thus forming a polycrystalline ring pattern because of their random orientations (Fig. 3c). The ‘orientation factor’ derived from the azimuthal profile of every single mesocrystal diffraction in the (311) plane decreased when polyacrylate was grafted onto the BNC surface, and reached a minimum value upon the injection of Mg^2+^ (Fig. 3d, Supplementary Fig. 9 and Supplementary Note 3). This decrease in the orientation factor indicates a discrepancy in the tiling of the BNCs during aggregative OA.

**Fig. 5:**
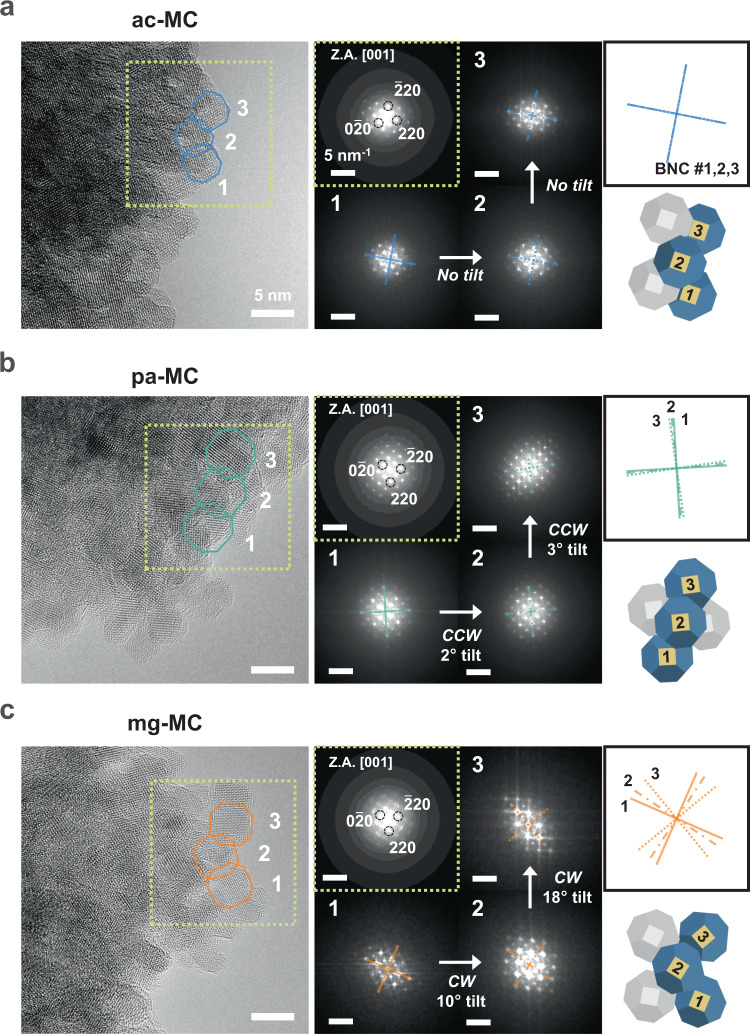
Local BNC alignment within a mesocrystal. **a**–**c** HR-TEM (left) images and FFT patterns (right) of ac-MCs with the ordered state (**a**), pa-MC with the intermediate state (**b**), and mg-MC with the disordered state (**c**). The yellow dotted boxes indicate the region with the [001] zone axis. Three FFT patterns were acquired from each BNC having the same numbering in the HR-TEM images. The tilt angle between adjacent BNCs was measured using the auxiliary crosses connecting {110} planes in each FFT pattern; solid lines: BNC #1, dash–single dotted line: BNC #2, and dotted line: BNC #3. Overlaid crosses and schematics were reconstructed based on each FFT pattern as a visual aid.

The BNCs were 4 nm truncated octahedrons predominantly formed by {100} and {111} facets (Supplementary Fig. 10). Atomic-resolution high-angle annular dark-field scanning transmission electron microscopy (HAADF-STEM), HR-TEM, and the corresponding FFT images obtained along the [001] and [011] zone axes indicated that the Fe_3_O_4_-truncated octahedron BNCs were single crystals with the inverse spinel structure, and Fe atoms (expectedly) occupied the octahedral and tetrahedral sites (Supplementary Fig. 10). We observed BNC tiling along the same [001] zone axis (Fig. 5a–c). In the ac-MCs, the BNCs completely packed together with coherent orientational ordering that minimised the {111} facets while forming eight {111}-to-{111} and four {100}-to-{100} facet-to-facet contacts per BNC (Fig. 5a). The pa-MCs exhibited the same tiling rule as the ac-MCs, but the adjacent BNCs were tilted by approximately 2°, resulting in a stretched spot in the collective FFT pattern (Fig. 5b and Supplementary Fig. 11). Upon adding Mg^2+^, adjacent BNCs randomly clustered with a tilt angle of 14°, making it difficult to determine a consistent tiling rule (Fig. 5c and Supplementary Fig. 11).

The degree of BNC alignment can influence grain formation in mesocrystals. In all samples, the BNCs measured via TEM were approximately 4 nm with similar morphologies, regardless of the type of ligand (Fig. 4b and Supplementary Fig. 10). However, the grain size measured via X-ray diffraction (XRD) decreased with the misalignment induced by polyacrylate and Mg^2+^ ions (Fig. 4b and Supplementary Fig. 12). When the reaction time was 10 h, the ac-MCs stopped growing after 3.5 h, and coarsening occurred between the BNCs thereafter, leading to the collapse of the nanogranular morphology and formation of larger grains (Supplementary Fig. 13). Interestingly, coarsening proceeded along the perfectly aligned region, and the crystallite sizes measured by XRD were maintained during coarsening (Fig. 2c and Supplementary Fig. 13). In the pa-MC cases, however, the BNCs were not coarsened, even at 10 h postreaction, and the nanogranular morphology was maintained because of the misalignment between the BNCs (Supplementary Fig. 13).

### Crystallographic-order-dependent magnetic property

**Fig. 6:**
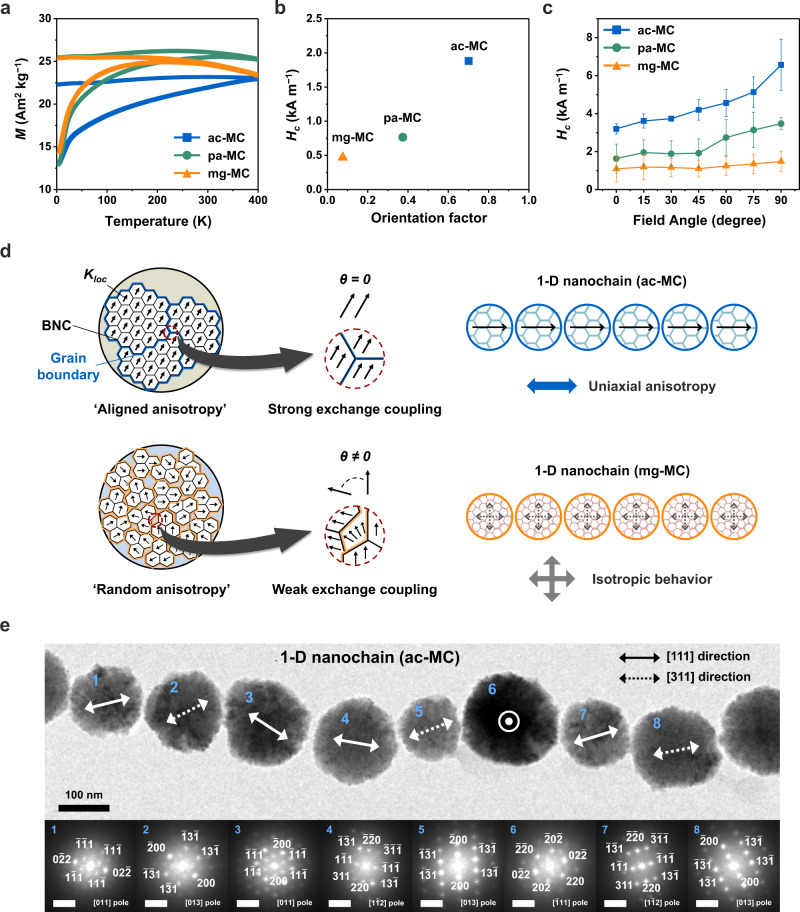
Magnetic properties of mesocrystals and their 1-D nanochains depending on the BNC alignment. **a** Zero-field-cooling (ZFC)/field-cooling (FC) magnetisation curves. **b** *H*_*c*_ plot of Fe_3_O_4_ mesocrystals depending on the crystallographic order in BNC alignment. **c** *H*_*c*_ behaviour of the nanochains as a function of the angle *θ* between the axial directions of the nanochains and the direction of the applied magnetic field. Each point is the average of three different measurements; the error bars denote the standard deviations. **d** Effects of inter-BNC magnetic interactions on the magnetic behaviour of mesocrystals and their nanochains. Single arrows indicate the local magnetocrystalline anisotropy. **e** BF-TEM image of a 1-D nanochain composed of Fe_3_O_4_ mesocrystals with aligned BNCs (ac-MCs). The ac-MCs are arranged in the direction of the magnetic field (160 kA m^−1^). The solid and dotted double arrows indicate the [111] and [311] directions of the Fe_3_O_4_ unit crystal derived from the FFT analysis. The scale bars in the FFT images represent 5 nm^–1^.

The degree of crystallographic ordering of BNCs within a mesocrystal can influence the inter-BNC magnetic coupling, thus resulting in a variation in the collective magnetic properties of the mesocrystal^29^. The saturation magnetisation (*M*_*s*_), blocking temperature (*T*_*B*_), and magnetic coercivity (*H*_*c*_) of the Fe_3_O_4_ mesocrystals decreased as the BNCs became misaligned (Fig. 6a, b, Supplementary Fig. 14 and Supplementary Table 4). The disordered BNC alignment in the mesocrystals represented superparamagnetic behaviour with *T*_*B*_ = 216 K (Fig. 6a). Given that Fe_3_O_4_ mesocrystals can be regarded as nanocrystalline systems composed of different grain sizes and orientations depending on the BNC alignment, their magnetic properties are imparted by the distribution of the magnetic anisotropy of grains and exchange coupling between grains, which can be expressed as $F=A{[\nabla m(r)]}^{2}+{K}_{loc}\{{[m(r)\cdot n(r)]}^{2}-\frac{1}{3}\}$. In this equation, *F*, *A*, *K*_*loc*_, *m*(*r*), and *n*(*r*) are the free energy, exchange stiffness, local anisotropy constant, local reduced magnetisation (*M*(*r*)/*M*_*s*_), and unit vector of the local easy axis, respectively (Fig. 6d)^19^. In the structurally identical region within the mesocrystal, local anisotropy formed in the <111> direction owing to the magnetocrystalline anisotropy of the inverse spinel structure of Fe_3_O_4_^30^. Furthermore, the magnetic moments of the grains were coupled to each other through exchange interactions on the scale of a few nanometres. Therefore, ac-MCs with BNCs that are aligned in the same direction can have the strongest anisotropy and exchange interactions, thereby increasing *M*_*s*_, *T*_*B*_, and *H*_*c*_. When the mesocrystals had disordered grains smaller than 10 nm because of BNC misalignment, the local anisotropy varied with the position, thus resulting in random anisotropy, attenuating the exchange coupling, and decreasing *M*_*s*_, *T*_*B*_, and *H*_*c*_^31,32^.

To examine how the interactions between mesocrystals depend on the BNC alignment, we arranged mesocrystals in a one-dimensional (1-D) nanochain under an external magnetic field of 160 kA m^−1^ (Fig. 6c, e and Supplementary Fig. 15). Interestingly, the ac-MCs aligned along the <111> easy axis of the magnetocrystalline anisotropy owing to the interaction anisotropy, which induces the uniaxial anisotropy of nanochains due to magnetostatic coupling between mesocrystals (Fig. 6e)^19,33^. In contrast, the mesocrystals with misaligned BNCs did not exhibit a particular orientation (Supplementary Fig. 15). We also explored the angular dependence of the *H*_*c*_ behaviour in the nanochains by varying the angle between the chain axis and magnetic field (Fig. 6c). The coercivity of the magnetic chains arranged in the <111> direction showed a pronounced dependency on this angle, with a minimum at *θ* = 0° and maximum at *θ* = 90°. This result agrees with the theoretical prediction of the chain-of-spheres model and magnetic chains formed by magnetotactic bacteria^33–35^. However, this angle dependency tended to diminish as the BNCs became disordered. Finally, the nanochains of mg-MCs showed an isotropic magnetic coercive force for all field angles, indicating that the magnetostatic interaction was not sufficiently strong to induce anisotropy along the nanochain axis; thus, the nanochain behaved like an isolated superparamagnetic mesocrystal (Fig. 6d).

### Driving force guiding BNC tiling rule

**Fig. 7:**
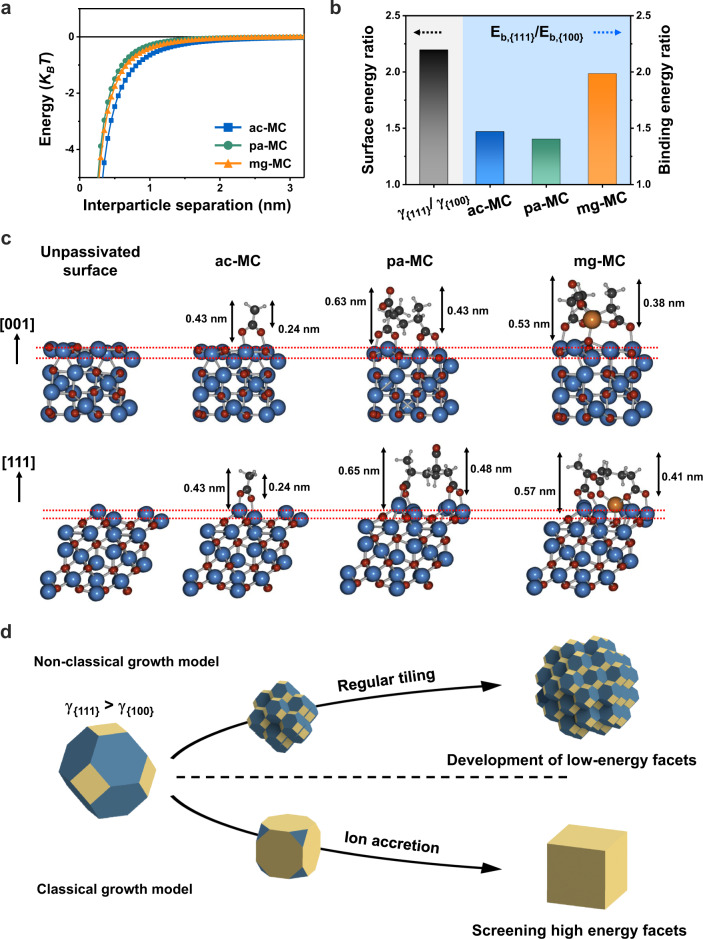
Driving force guiding the variation in the BNC alignment during OA. **a** Theoretical model of the pair interaction potential between BNCs as a function of the inter-BNC distance calculated by the extended DLVO theory, considering van der Waals attraction, electrostatic repulsion, and magnetic dipole–dipole interactions. **b** Surface energy ratio of unpassivated BNCs and ratio of the binding energies of surface ligands for the {111} and {100} facets, as calculated by DFT. **c** Side view of the BNC surface conformation and surface ligand simulated by DFT calculations after relaxation. Relaxed slab structure of BNC before and after surface ligand coordination on the {001} and {111} facets. The blue, black, white, red, and orange spheres indicate Fe, C, H, O, and Mg atoms, respectively. The red dotted auxiliary lines illustrate the surface distortion relative to the unpassivated Fe_3_O_4_ surface. **d** Classical facet overgrowth and nonclassical regular tiling processes.

The driving force guiding the assembly of colloidal nanocrystals is generally explained by interparticle interactions using the DLVO theory (Fig. 7a and Supplementary Note 5)^20^. This pair interaction potential shows the energy barrier balanced by the attractive and repulsive forces between BNCs and suggests their collision kinetics. The nanoscale interactions occurring in our system can be described by van der Waals, electrostatic, and magnetic dipole–dipole forces. In our system, the Debye screening lengths (*κ*^−1^) were estimated to be 0.64, 0.65, and 0.54 nm for the ac-MCs, pa-MCs, and mg-MCs, respectively. Therefore, the surface-charge-induced repulsion had little effect on the overall interaction potential. Furthermore, considering the superparamagnetic BNCs commensurate with extremely small sizes (less than 10 nm), magnetic Keesom interactions can be ignored in our case^3^. Therefore, the attractions between BNCs dominated the overall potential through the van der Waals interaction, thereby forming a barrierless interaction potential regardless of the type of surface ligand and leading to preferential assembly between the BNCs (Fig. 7a). Despite the generality of the DLVO theory, this surface-ligand-induced variation in the BNC alignment cannot be rationalised based on traditional pairwise interactions.

To better understand the factors governing alignment variation induced by different ligands, we examined the surface energy and ligand binding energy considering the ligand configurations on the {100}, {110}, and {111} planes by performing DFT calculations (Fig. 7b, c, Supplementary Figs. 16–18, and Supplementary Note 5). The surface energies of unpassivated Fe_3_O_4_ for {100}, {110}, and {111} were 0.51, 4.24, and 1.12 eV nm^–2^, respectively (Table 1). Consistent with the significantly high energy of the {110} facet according to the DFT calculations, experimentally observing the {110} facets via TEM was more challenging than observing the {100} and {111} facets (Supplementary Fig. 10).

Concurrently, the binding affinity of all ligands to the {111} facets is also more substantial than that to the {100} facets, which can relax the anisotropic surface energies (*γ*_(111)_ – *γ*_(100)_ > 0) by passivating the dangling bonds (Table 1). For acetate, the binding energy ratio of the {111} and {100} facets was 1.46 (*E*_*b*_,_(111)_/*E*_*b*,(100)_), which is insufficient to alleviate the surface energy anisotropy, resulting in a crystallographically ordered BNC alignment that minimises the surface energy by eliminating the {111} facets rather than the {100} ones (Fig. 7b). With polyacrylate, the binding energy to all facets notably increased, although the *E*_*b*_,_(111)_/*E*_*b*,(100)_ ratio was 1.40, thereby maintaining the regularity of the acetate-like tiling rule (Fig. 7b). Meanwhile, the carboxylate of the surface ligands coordinated by bidentate bridging on each BNC facet distorted the positions of the Fe and O atoms and created a zigzag topography, which was prominent on the polyacrylate-grafted surfaces (Fig. 7c). This topography hindered the perfect coherence in the attachment between facets and slightly decreased the orientation factor, as determined in the TEM observations. In the mg-MCs, Mg^2+^ ions could bridge the unbound free carboxylate of polyacrylate and O on the Fe_3_O_4_ surface, which agreed with the decreased electrokinetic potential (Fig. 7c and Supplementary Fig. 7). The Mg^2+^ion-induced distortion in the configuration of acrylate coordination on Fe_3_O_4_ increased the difference in binding energy between the {111} and {100} facets, with an *E*_*b*_,_(111)_/*E*_*b*,(100)_ ratio of 1.98, thus suppressing the anisotropic surface energies and disorder in the tiling rules of BNC attachment (Fig. 7b).

Generic facet formation and the growth of single-crystal nanoparticles were driven by surface energy minimisation following the Wulff construction in the classical theory (Fig. 7d)^36,37^. Considering that the volume free energy is negligible during the aggregative OA process accompanied by a constant number of BNCs, the BNC tiling rule to form thermodynamically stable aggregates is governed by the surface energy. These results indicate that the disorder–order transitions in the BNC alignment were induced by surface ligand coordination, which resulted in variations in the anisotropic surface energy and surface distortion of the BNCs. Consequently, similar to self-assembly, when BNCs have anisotropy in surface energy, the assembled structure follows trends that expose low-energy facets or eliminate high-energy facets^38,39^.

**Table 1:** Calculated surface energies and ligand binding energies for the different facets of Fe_3_O_4_.

Facet	Surface energy (eV nm^–2^)	Binding energy (eV per molecule)		
		ac-MC	pa-MC	mg-MC
{100}	0.51	3.48	8.19	7.49
{110}	4.24	3.40	23.64	11.81
{111}	1.12	5.11	11.49	14.86

## Discussion

In summary, the crystallisation and growth of Fe_3_O_4_ mesocrystals can be chemically controlled into a single pathway via OA. Upon OA, the coordination of different surface ligands, namely, acetate, polyacrylate, and Mg^2+^-adsorbed polyacrylate, altered the crystallographic order of the BNC tiling rule, thereby producing mesocrystals with different grain structures. BNC alignment significantly affected the inter-BNC and intermesocrystal magnetic interactions (via exchange coupling and dipole–dipole interactions, respectively), resulting in different collective magnetic behaviours in individual Fe_3_O_4_ mesocrystals and their magnetic chains. We then revealed that the tuneable alignment of BNCs is guided by the facet-dependent surface energy of BNCs and changes in the surface energy after ligand coordination. These results help elucidate the driving force guiding the OA of Fe_3_O_4_ and a fundamental magnetic model for mesocrystal assembly with different microstructural orders. This work provides a starting point for the grain engineering of nanocrystalline materials based on control of the crystallisation process in natural and synthetic systems, as well as relevant practical applications.

## Methods

### Chemicals

Iron chloride hexahydrate (FeCl_3_·6H_2_O, >97%, Sigma-Aldrich, Korea), NaOAC (>98.5%, Sigma-Aldrich, Republic of Korea), sodium acrylate (NaAc, 97%, Sigma-Aldrich, Republic of Korea), magnesium chloride hexahydrate (MgCl_2_·6H_2_O, >99%, Sigma-Aldrich, Republic of Korea), and ethylene glycol ((CH_2_OH)_2_, >99%, Alfa Aesar, Republic of Korea) were used as received without further purification. Deionized water was obtained using a Millipore Direct-Q UV 3.

### Chemical control of crystallisation pathways of multigrain Fe_3_O_4_ mesocrystals

We used a modified polyol method to synthesise the Fe_3_O_4_ mesocrystals. This method has been extensively employed to produce metal and metal oxide mesocrystals of various sizes^40^. We employed iron chloride hexahydrate (FeCl_3_∙6H_2_O) as a Fe^3+^ ion precursor, and used NaOAC and deionised water (Millipore Direct-Q UV 3) as the hydroxyl ion sources. Ethylene glycol was used as a solvent and reducing agent. We prepared solution 1 by dissolving FeCl_3_∙6H_2_O in deionised water, and solution 2 by dissolving NaOAC in 50 mL of ethylene glycol under gentle sonication for 10 min. We adjusted the concentrations of FeCl_3_∙6H_2_O and NaOAc in each solution to analyse the changes in the crystallisation pathways according to the OH^−^/Fe^3+^ ratio. To analyse the effects of NaOAC on the crystallisation pathway, 6, 10, and 15 mmol of NaOAC were dissolved in 50 mL of ethylene glycol for solution 2, whereas 2 mmol of FeCl_3_∙6H_2_O was dissolved in 150 mmol of H_2_O for solution 1. To produce mesocrystals only through pathway 1, we prepared solution 1 with 2 mmol of FeCl_3_∙6H_2_O in 150 mmol of H_2_O, and solution 2 containing 6 mmol of NaOAC in 50 mL of ethylene glycol. To produce mesocrystals only through pathway 2, 1 mmol of FeCl_3_∙6H_2_O was dissolved in 200 mmol of H_2_O for solution 1, and 3 mmol of NaOAC was dissolved in 50 mL of ethylene glycol. In a typical synthesis process, after preparing solutions 1 and 2 with the appropriate concentrations, they were mixed under vigorous stirring to form a turbid, yellow-brown solution, followed by refluxing for 8 h at 200 °C. During refluxing, this solution gradually turned reddish-brown and then black. After cooling to room temperature, the black sediment was washed more than five times using ethanol to eliminate any by-products. To observe the mesocrystal formation, the experiments were stopped in 30 min or 1 h intervals, and the samples were stored at 4 °C in a refrigerator. The reaction time varied depending on the crystallisation pathway. With pathway 1, in which the mesocrystals grew via OA, the growth of Fe_3_O_4_ mesocrystals was saturated after 3.5 h of refluxing, followed by grain coarsening between BNCs.

### Acetate-coordinated Fe_3_O_4_ mesocrystals

These mesocrystals were prepared in the same way as above, but different amounts of the precursors were used to prepare solutions 1 and 2, as follows: 2 mmol of FeCl_3_·6H_2_O and 150 mmol H_2_O were utilised for solution 1, and 6 mmol of NaOAC and 50 mL of ethylene glycol were used for solution 2. The mixture of the solutions was heated to 200 °C at a heating rate of 10 °C min^−1^ and then refluxed over 3.5 h at 200 °C. Without additional moieties, acetate was attached to the BNC surface because NaOAC was used as the OH^–^ source in the original synthesis method. To analyse the effects of the Fe^3+^ precursor on the mesocrystal size, we increased the content of FeCl_3_·6H_2_O from 1 to 3 mmol in 0.5 mmol intervals while fixing [NaOAC]/[FeCl_3_·6H_2_O], H_2_O, and ethylene glycol to 3, 150 mmol, and 50 mL, respectively.

### Polyacrylate-coordinated Fe_3_O_4_ mesocrystals with and without Mg^2+^ adsorption

We prepared these mesocrystals in the same manner as above but used different amounts of the precursors to prepare solutions 1 and 2 as follows: 2 mmol of FeCl_3_·6H_2_O and 150 mmol H_2_O were employed for solution 1, and 6 mmol of NaOAC, 0.5 mmol of NaAc, and 50 mL of ethylene glycol were utilised for solution 2. The mixture of the solutions was heated to 200 °C at a heating rate of 2 °C min^−1^ before refluxing over 3.5 h at 200 °C. We added NaAc to the initial reacting solution 2; the low heating rate enabled the acrylate molecules to be polymerised into polyacrylate and coordinated on the Fe sites of the BNC surface^41^. To adsorb Mg^2+^ onto the polyacrylate-grafted surface, we injected 1 mmol of MgCl_2_·6H_2_O dissolved in 10 mmol of H_2_O into the reaction mixture when the temperature reached 200 °C.

### Microstructural characterisation

We analysed Fe_3_O_4_ mesocrystals synthesised with various chemical contents to control the crystallisation pathways by performing analytical TEM (JEOL, JEM-2100F) at 200 kV. The formation process at different reaction times and BNC alignment variation depending on the surface ligands were investigated by double *C*_*s*_ and monochromated TEM (FEI, TITAN) at 300 kV. We also examined the compositions of the mesocrystals coordinated by Mg^2+^-adsorbed polyacrylate by performing analytical TEM (FEI, Talos F200X) at 200 kV. To observe the formation process of Fe_3_O_4_ mesocrystals while preserving the samples, we utilised a crude solution for sampling on a TEM grid without washing or dilution. Specifically, we dropped 7 μL of the solution onto a C-coated Cu grid or a Quantifoil holey C grid. To sufficiently adsorb the intermediate and mesocrystal into the grid, we allowed the solutions to sit for approximately 1 h in a vacuum desiccator and removed the remaining solution through filter paper. The samples for the SAED, HR-TEM, FFT, HAADF-STEM, EELS, and EDS elemental mapping analyses were prepared by diluting the samples after washing with absolute ethanol and then placing a drop of the sample solution on a C-coated Cu grid or a Quantifoil holey C grid. We measured the sizes of *n* > 100 Fe_3_O_4_ mesocrystals and *n* > 50 BNCs from the TEM images and fitted the data using Gaussian distributions. The azimuthal profile and *d-*spacing of the SAED pattern were analysed using the Gatan DigitalMicrograph, including the DiffTools script. We determined the crystal structure and grain size of the Fe_3_O_4_ mesocrystals by performing powder XRD (PANalytical, X’Pert ProMPD) with a Cu Kα radiation source (*λ* = 1.5406 Å) at the Korea Basic Science Institute, Seoul Western Center. We recorded XRD patterns in a *θ*–2*θ* geometry from 20° to 80° using a 45 kV and 40 mA tube. The average grain size was calculated from the (311) main peak of Fe_3_O_4_ using the Scherrer equation after fitting the profiles of the samples with a pseudo-Voigt function and correcting for instrumental broadening.

### Surface characterisation

The amount of surface ligands on BNCs was analysed by conducting TG-DTA (TA Instrument, SDT Q600) under Ar flowing at 100 mL min^−1^. Vacuum-dried samples were mounted onto a holder and heated from room temperature to 500 °C at a ramp rate of 10 °C min^−1^. Considering the weight change (%) and temperature differences (°C mg^−1^), the ligand densities of acetate, polyacrylate, and Mg^2+^-adsorbed polyacrylate were quantified using the following equation: ${\sigma }_{{{{{{\rm{TGA}}}}}}}=\frac{\frac{{{{{{\rm{wt}}}}}}{ \% }_{{{{{{\rm{polymer}}}}}}}}{{{{{{\rm{wt}}}}}}{ \% }_{{{{{{\rm{{Fe}}}}}_{3}{O}_{4}}}}}{\rho }_{{{{{{\rm{{Fe}}}}}_{3}{O}_{4}}}}\frac{4}{3}\pi {r}_{{{{{{\rm{{Fe}}}}}_{3}{O}_{4}}}}^{3}{N}_{A}}{{M}_{n}4\pi {r}_{{{{{{\rm{{Fe}}}}}_{3}{O}_{4}}}}^{2}}$, where *σ* is the ligand density, wt% is the relative mass of each element in the temperature range of 200–400 °C, *ρ* is the density of Fe_3_O_4_ (5.2 g cm^–3^), *N*_*A*_ is Avogadro’s number, *M*_*n*_ is the molecular weight of a surface ligand unit, and *r* is the BNC radius^42^. We recorded FT-IR spectra (Thermo Scientific, Nicolet iS50) of the Fe_3_O_4_ mesocrystals with different surface ligands in the ATR mode with a diamond crystal window. Vacuum-dried samples were mounted onto the ATR crystal and then measured at a resolution of 2 cm^−1^ in the range of 4000–400 cm^−1^. We collected XPS (Thermo Scientific, K-Alpha^+^) profiles with a monochromated Al *Kα* X-ray source operating at 1486.6 eV. A survey scan including the photoemission features from all elements was collected with a pass energy of 200 eV in 1.0 eV intervals, and detailed scans of narrow elemental regions (Fe 2*p*, O 1*s*, C 1*s*, and Mg 1*s*) were recorded at 50 eV in 0.1 eV intervals. All energy-loss regions in the narrow scan spectra were fitted using Shirley background functions. We performed atomic absorption spectrometry (Thermo Scientific, iCE 3500) and inductively coupled plasma optical-emission spectrometry (Thermo Scientific, iCAP 6500 Duo) to determine the relative chemical compositions of Mg^2+^ and Fe^3+^/Fe^2+^, respectively, in the mesocrystals coordinated with Mg^2+^-adsorbed polyacrylate. Dynamic light scattering and zeta-potential measurements were conducted using the Zetasizer Nano ZS90, Malvern Instruments, with diluted samples dispersed in 0.5 mL of deionised water.

### Magnetic property characterisation

We analysed the magnetic properties of the powder samples with different crystallographic orders by measuring the magnetisation curves *M*(*H*) and zero-field-cooling (ZFC)/field-cooling (FC) curves using a vibrating sample magnetometer (Microsense, EV9) and a physical property measurement system (PPMS, Quantum Design, PPMS-14) equipped with a vibrating sample magnetometer. The magnetisation curves *M*(*H*) of Fe_3_O_4_ mesocrystals with different BNC alignments were measured at 300 K using an applied field of up to 1593 kA m^−1^. We performed the ZFC/FC measurements using the PPMS in the sweep mode with a temperature variation rate of 10 K min^−1^. In detail, all samples were cooled to 4 K from room temperature without applying an external field (*H*_*ex*_ = 0), and the magnetisation of the mesocrystals was collected with increasing temperature up to 400 K in a field of 40 kA m^−1^ (ZFC). Subsequently, the samples were cooled to 4 K again under the same external field strength, and the magnetisation was measured by the same process. To examine the angular dependence of the *H*_*c*_ behaviours of 1-D Fe_3_O_4_ nanochains, we measured the magnetisation curves *M*(*H*) of each nanochain with different BNC alignments using the EV9 vibrating sample magnetometer equipped with a rotating holder. Specifically, we dispersed different types of Fe_3_O_4_ mesocrystals with different BNC alignments in ethanol at a concentration of 0.01 mg mL^−1^, and then dropped 10 μL of the solution on a 6.4 × 6.4 mm^2^ oxidised Si wafer substrate under an external magnetic field of 160 kA m^−1^. To ensure that only mesocrystal–mesocrystal magnetic interactions in the nanochain affected the magnetisation, we maintained a large inter-nanochain distance, and ensured that each nanochain was composed of only one row of mesocrystals, thus minimising nanochain–nanochain magnetic interactions.

## Supplementary information

Supplementary Information

## Data Availability

The data that support the findings of this study are available from the corresponding author upon request. Source Data are provided with this paper.

## Source data

Source Data
