# Supplementary material for: Surface-ligand-induced crystallographic disorder–order transition in oriented attachment for the tuneable assembly of mesocrystals
Source: Nat Commun. 2022 Mar 3;13:1144. doi: 10.1038/s41467-022-28830-7 (PMC8894404; doi:10.1038/s41467-022-28830-7)
Supplement: Supplementary file 1 — Supplementary Information [file 41467_2022_28830_MOESM1_ESM.pdf]

Supplementary Information

Surface-ligand-induced crystallographic disorder–order transition in oriented attachment for the tuneable assembly of mesocrystals

Bum Chul Park[1,2 †], Min Jun Ko[1 †], Young Kwang Kim[3], Gyu Won Kim[1], Myeong Soo Kim[1,4], Thomas Myeongseok Koo[1], Hong En Fu[1], and Young Keun Kim[1,2,4 *]

[1]Department of Materials Science and Engineering, Korea University, Seoul 02841, Republic of Korea
[2]Brain Korea Center for Smart Materials and Devices, Korea University, Seoul 02841, Republic of Korea
[3]Virtual Lab Inc., Seoul 04799, Republic of Korea
[4]Institute of High Technology Materials and Devices, Korea University, Seoul 02841, Republic of Korea

*E-mail address: ykim97@korea.ac.kr (Y. K. Kim).
†These authors contributed equally to this study.

Supplementary Note 1. Crystallisation pathway control

1. Chemical control of crystallisation pathways

We synthesised Fe_3O_4 mesocrystals consisting of assemblies of nanometric building-block nanocrystals (BNCs) using a modified polyol method.[1] In this synthesis reaction, the Fe_3O_4 phase does not form directly from ions, but rather forms through a stepwise transformation preceded by several metastable intermediates, following the Ostwald step rules.[2] Ferrihydrite, ferric oxyhydroxides (lepidocrocite γ-FeOOH, and goethite α-FeOOH), and green rusts are frequently observed as metastable intermediates for the Fe_3O_4 phase.[3–8] As these intermediates have similar solubility (10^{-7}–10^{-8} M), the type of intermediate can vary depending on the chemical environment (e.g., the precursor chemical content ratio or impurities).[9–12] Previously, we examined the effects of intermediate polymorphism on the microstructures (crystallite sizes) of Fe_3O_4 mesocrystals and discovered two crystallisation pathways.[1] When Fe_3O_4 mesocrystals are directly transformed from lepidocrocite, the mesocrystals grow via oriented attachment (OA), producing small crystallites (pathway 1). If the Fe_3O_4 mesocrystals grow immediately after the transformation of lepidocrocite into goethite, the mesocrystals are crystallised via interfacial growth (dissolution and re-precipitation), leading to large crystallites (pathway 2). These two crystallisation pathways coexist competitively but are non-concurrent during the chemical reaction; thus, they are distinguishable because they are initiated at different reaction stages.

In this study, we precisely controlled Fe_3O_4 mesocrystals to grow through a single pathway by regulating the chemical conditions. We examined the ratio of the crystallisation pathways depending on the contents of the hydroxyl ion sources (sodium acetate (NaOAC) and H_2O) and the Fe^{3+} ion precursor ($FeCl_3 \cdot 6H_2O$), which are typical chemicals in the modified polyol method. The amount of ethylene glycol, which acts as both a solvent and reducing agent, was fixed at 50 mL. The crystallisation pathways should change according to the reaction environment, such as the Fe^{3+}:OH^- ratio and excess Fe^{3+} ions that can be reduced to Fe^{2+} by ethylene glycol. We identified the conditions under which Fe_3O_4 mesocrystals grow through either pathway 1 or 2 when the H_2O content was controlled and the chemical content ratio of $FeCl_3$:NaOAC was fixed at 1:3 (Supplementary Fig. 4). Again here, the two pathways were clearly distinguishable because they started at different reaction stages, as in our previous study. The transmission electron microscopy (TEM) images in Supplementary Fig. 4 show that in

pathway 1, the Fe_3O_4 mesocrystals formed in the nanocrystalline intermediate just after $t = 0.5$ h, and all the intermediates were consumed before $t = 3.5$ h. However, in pathway 2, the Fe_3O_4 mesocrystals began to form from the tubular intermediate after $t = 3$ h, and the transformation terminated at $t = 8$ h.

Based on these results, we examined the effects of NaOAC on the crystallisation pathways by increasing the NaOAC content from 6 to 15 mmol while fixing the $FeCl_3 \cdot 6H_2O$ (2 mmol) and H_2O (150 mmol) contents (Supplementary Fig. 3). As the NaOAC concentration increased, pathway 1 gradually became dominant. Finally, the Fe_3O_4 mesocrystals crystallised only through pathway 1 when they were synthesised using 2 mmol $FeCl_3 \cdot 6H_2O$, 6 mmol NaOAC, and 150 mmol H_2O.

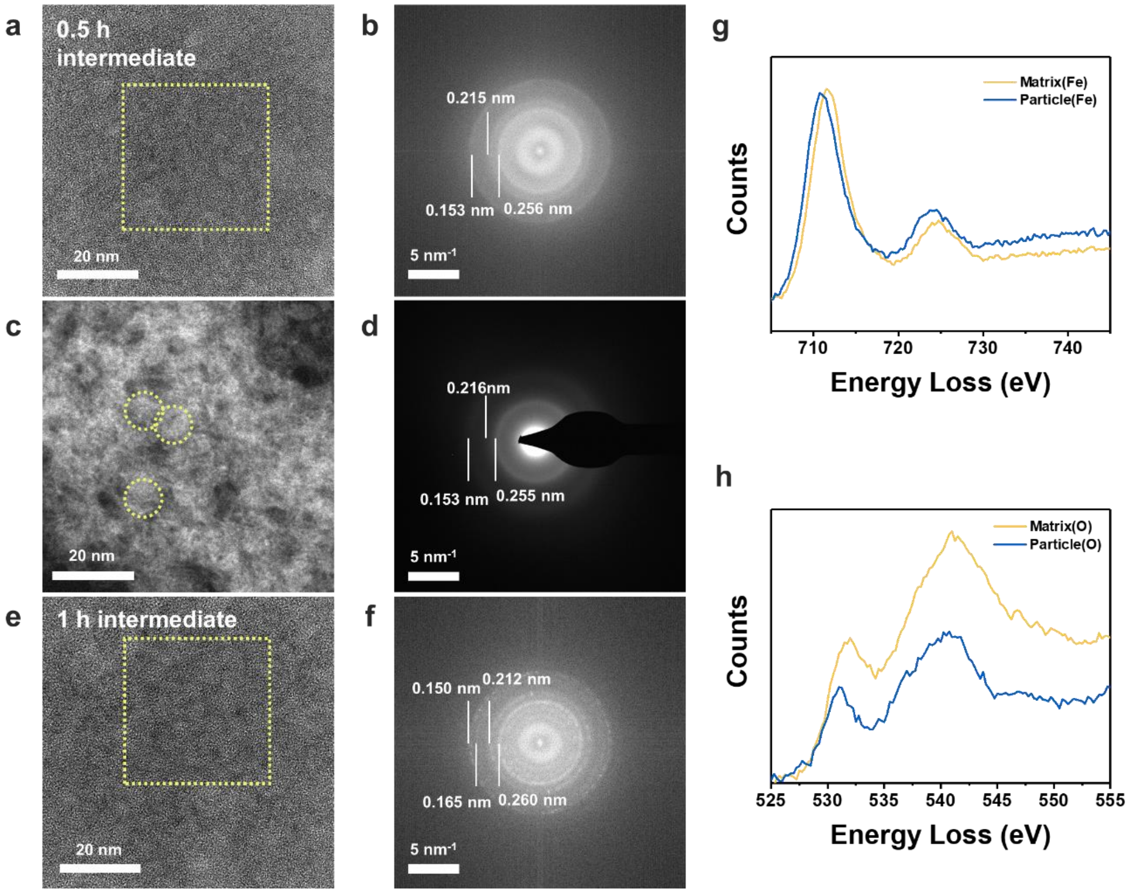

Supplementary Fig. 1 | Characterisation of intermediate phases. **a–f,** TEM images and corresponding SAED patterns of as-synthesised samples with reaction times of 0.5 h (**a–d**) and 1 h (**e, f**). **a**, BF-TEM image of poorly crystalline (nanocrystalline) ferric oxyhydroxide intermediate. **b**, FFT pattern acquired from the yellow dotted box in (**a**), representing diffused ring patterns with three *d*-spacings of 0.26, 0.21, and 0.15 nm. **c**, STEM image of ferric oxyhydroxide intermediate, wherein 3–4 nm spherical particulates are indicated by yellow dotted circles. **d**, SAED pattern collected from the intermediate formed at 0.5 h, which corresponds well with the FFT pattern in (**b**). **e**, Poorly crystalline ferric oxyhydroxide observed at 1 h. **f**, FFT pattern converted from the yellow dotted box in (**e**). **g**, **h**, EELS profiles of intermediate and Fe_3O_4 mesocrystals after 1.5 h of reaction; Fe $L_{2,3}$-edge level (**g**), O *K*-edge level (**h**). Interestingly, the peaks shift slightly towards lower energies in both spectra, suggesting that the intermediate is in the ferric state (Fe^{3+}) and the Fe_3O_4 mesocrystal is in a partially reduced state (Fe^{3+} and Fe^{2+}).

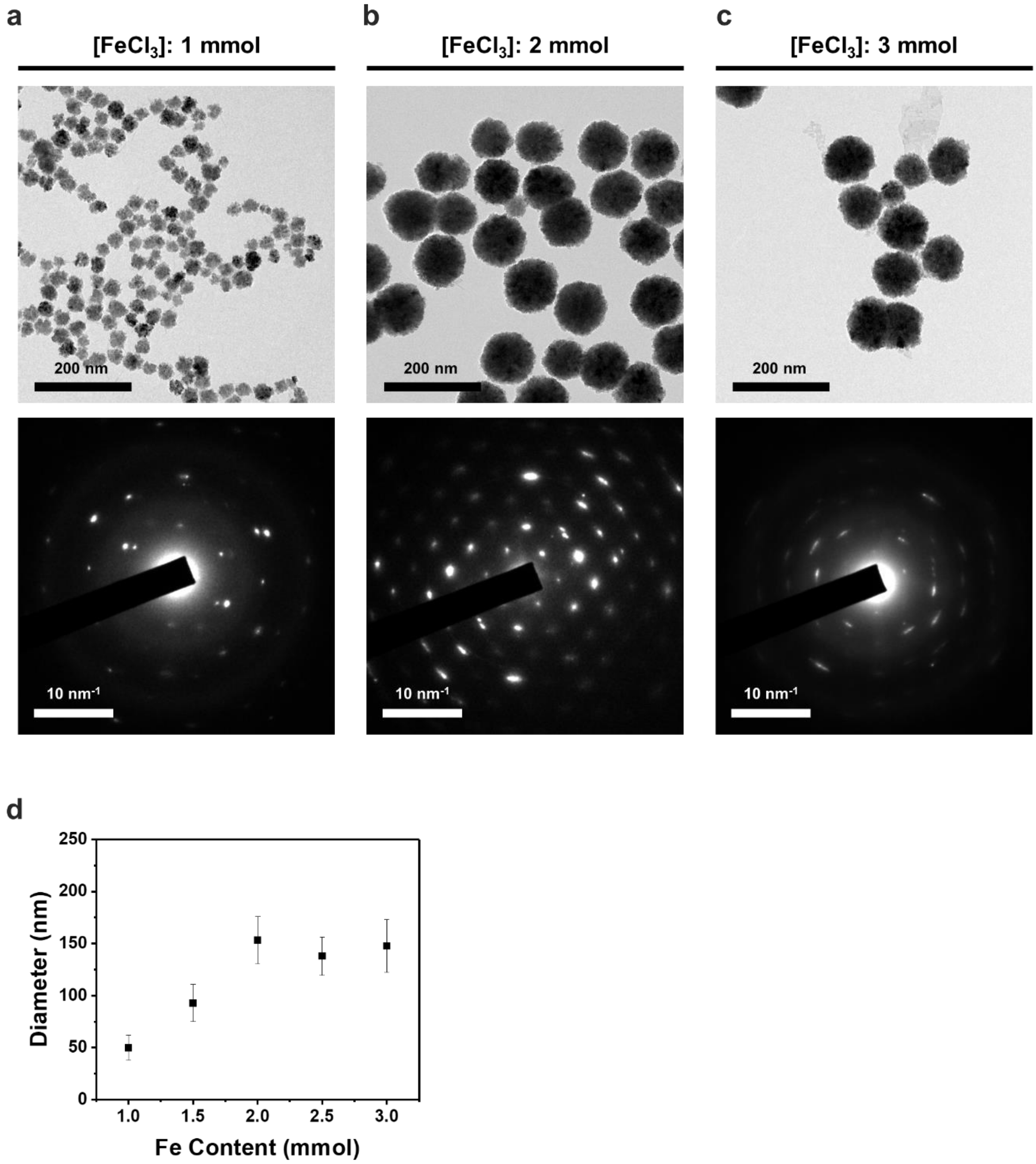

Supplementary Fig. 2 | Effect of Fe precursor on the Fe_3O_4 mesocrystal size. a–c, Representative TEM images and single-particle SAED patterns of Fe_3O_4 mesocrystals synthesised with the [NaOAC]/[$FeCl_3$] content fixed at 3 and different $FeCl_3$ contents: 1 mmol (**a**), 2 mmol (**b**), and 3 mmol (**c**). The SAED patterns were acquired from a single mesocrystal in each sample to demonstrate the crystallographically aligned BNCs. **d**, Average diameter of the Fe_3O_4 mesocrystal as a function of the Fe precursor content. The data points are the average values from n = 100; the error bars indicate the standard deviations.

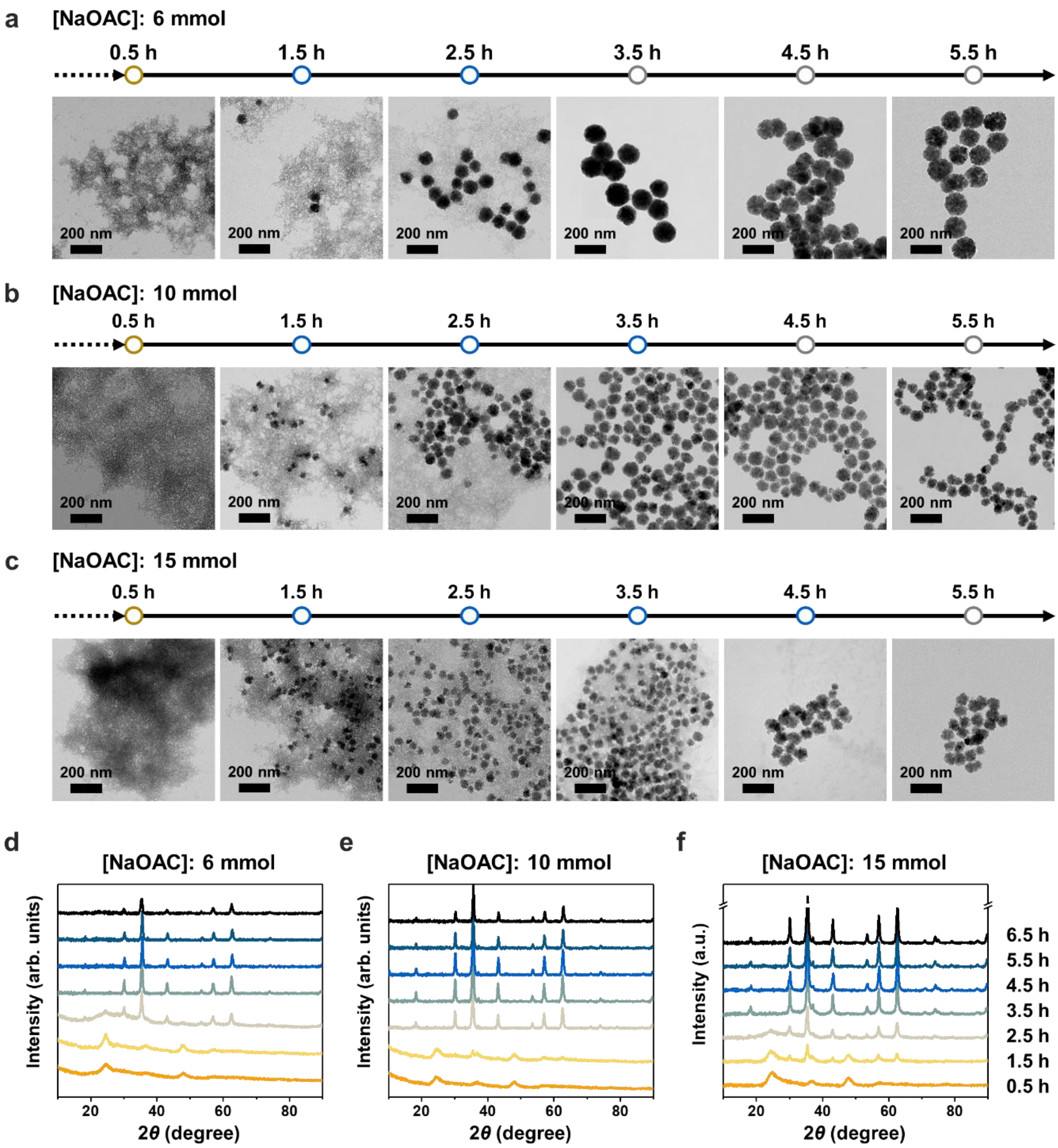

Supplementary Fig. 3 | Effects of NaOAC content on the crystallisation pathway. a–c, Sequential observation of different crystallisation pathways of Fe_3O_4 mesocrystals synthesised with different precursor ratios: $FeCl_3$:NaOAC:H_2O = 2:6:150 mmol (**a**), 2:10:150 mmol (**b**), and 2:15:150 mmol (**c**). The other parameters, including the amount of ethylene glycol, refluxing temperature, and reaction time, were constant. The circles on the timeline of the entire reaction indicate the phases appearing at each time, namely, the intermediate (yellow), Fe_3O_4 phase with intermediate (blue), and coarsened Fe_3O_4 phase (grey). **d–f**, XRD pattern under each chemical condition: $FeCl_3$:NaOAC:H_2O = 2:6:150 mmol (**d**), 2:10:150 mmol (**e**), and 2:15:150 mmol (**f**). As the NaOAC content decreases, crystallisation progresses more rapidly, i.e., the Fe oxyhydroxide intermediates more rapidly transform into crystalline magnetite mesocrystals.

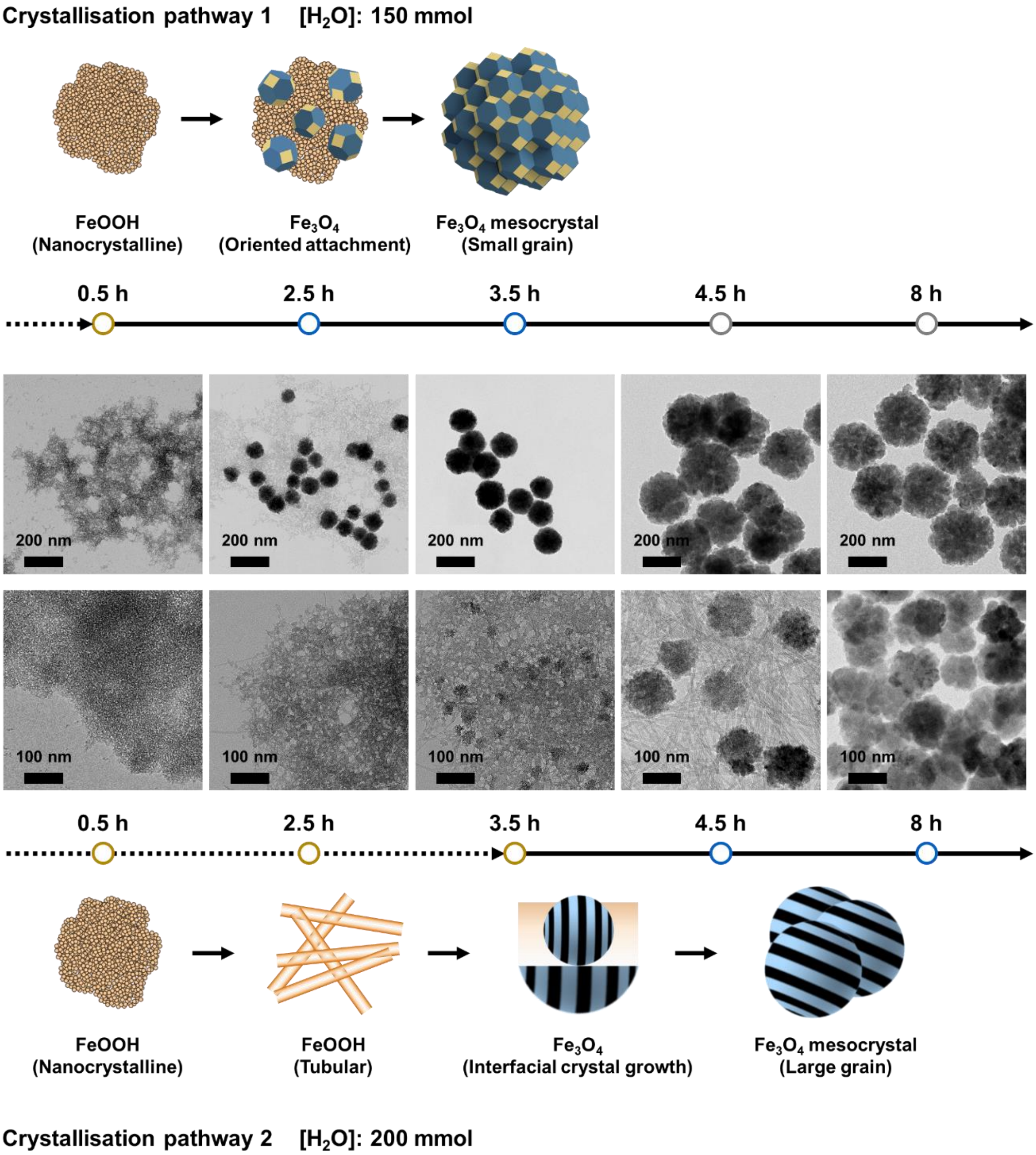

Supplementary Fig. 4 | Effects of H_2O content on the crystallisation pathway. a and b, Sequential observations of different crystallisation pathways for the Fe_3O_4 mesocrystals with H_2O contents of 150 mmol (**a**) and 200 mmol (**b**) and the NaOAC/$FeCl_3$ ratio fixed at 3. The TEM images and schematic demonstrate that the two different crystallisation pathways are controlled by the chemical content and are responsible for the growth of Fe_3O_4 mesocrystals, but they initiate asynchronously, i.e., the crystallisation pathways are dissimilar. The circles on the timeline of the entire reaction indicate the phases appearing at each time as nanocrystalline intermediates (yellow), tubular intermediate (pink), the Fe_3O_4 phase with the intermediate (blue), and the coarsened Fe_3O_4 phase (grey). Supplementary Note 1 describes in detail how the crystallisation process is controlled.

2. Kinetics of crystallisation pathway via OA

We examined the growth kinetics of a sample synthesised with a chemical ratio of $FeCl_3 \cdot 6H_2O$:NaOAC:H_2O = 2:6:150 mmol, which was crystallised only through pathway 1. The Fe_3O_4 mesocrystals no longer grew after 3.5 h of reaction (Fig. 2c). To gain further insights into the crystallisation mechanism of pathway 1, we analysed the growth kinetics of Fe_3O_4 mesocrystals using the Johnson–Mehl–Avrami–Kolmogorov (JMAK) model (Supplementary Fig. 5). This model helps explain the mechanisms of nucleation and growth in solid-state phase transformations and polymorphic transformations under isothermal conditions. Fe_3O_4 mesocrystals grow while consuming lepidocrocite as a precursor; thus, the JMAK model can be applied to our system, as expressed in the following equations:

$$f = 1 - \exp\left[-(kt)^n\right] \qquad (1)$$

$$\frac{V_t}{V_f} = 1 - \exp\left[-(kt)^n\right] \qquad (2)$$

$$\frac{\frac{4}{3}\pi {r_{t_{trans,pathway1}}}^3}{\frac{4}{3}\pi {r_1}^3} = 1 - \exp\left[-\left(kt_{trans,pathway1}\right)^n\right], \qquad (3)$$

where f is the volume fraction of the transformed Fe_3O_4 mesocrystal, t is the reaction time, k is the rate constant, and n is the Avrami exponent.[13–15] Fe_3O_4 growth consumes all intermediate phases; thus, we can express the volume fraction f as V_t/V_f, where V_t is the volume of Fe_3O_4 mesocrystals at reaction time t, and V_f is the saturated volume of Fe_3O_4 mesocrystals. The volume at each reaction time was calculated using the radius measured from the TEM images. The experimental data describing the growth kinetics of Fe_3O_4 mesocrystals were well fitted by the prediction of the JMAK model (Supplementary Fig. 5). The Avrami exponent, which provides information about the mechanism of phase transformation, was estimated to be 2.75, which was in good agreement with the reported value for the growth of Fe_3O_4 mesocrystals via OA.[1] This exponent can be written as $n = a + (b \times c)$, where a is the time-dependent nucleation rate ($a > 0$), b is the dimensionality of the growth ($0 < b < 3$), and c is the growth mode (c = 0.5 or 1).[14,15] Based on TEM analysis (Fig. 1), we identified that the Fe_3O_4 mesocrystals grew spherically in three dimensions during OA (b = 3). The c value was determined to be either 0.5 or 1 depending on the growth mode, corresponding to volume diffusion growth and interface-movement-controlled growth, respectively. During OA, the BNCs approached and attached to each other through diffusion, which can be inferred as c = 0.5. Therefore, the a value can be estimated to be approximately 1.2–1.3, where $a > 1$ suggests an increasing nucleation rate for BNC formation in the intermediate matrix and a collision between BNCs.

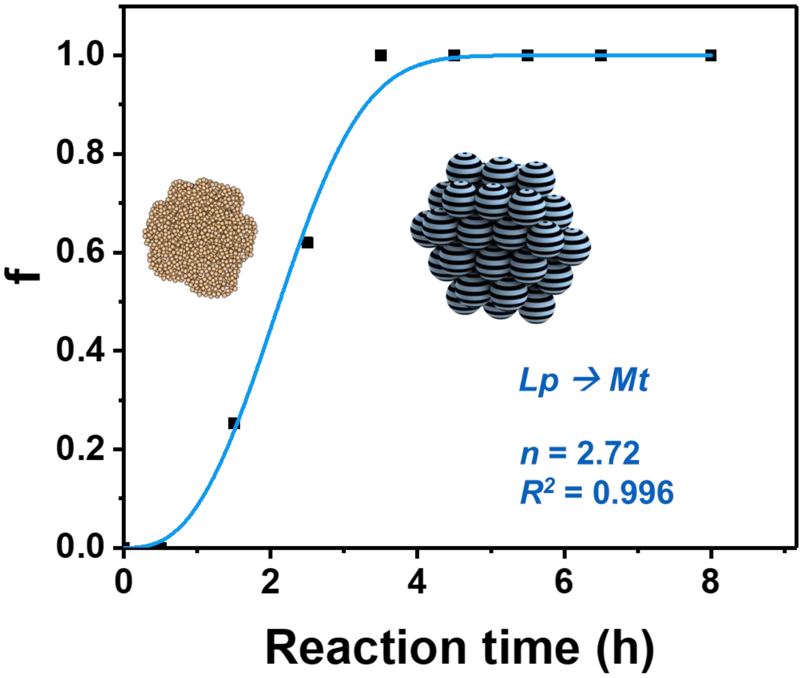

Supplementary Fig. 5 | Growth kinetics analysis of Fe_3O_4 mesocrystals using the JMAK model. Plot of the volume fraction (*f*) of the transformed Fe_3O_4 (magnetite, Mt) mesocrystals from iron oxyhydroxide intermediates (lepidocrocite, Lp) as a function of reaction time. The values of n and R^2 indicate the Avrami exponent and coefficient of determination, respectively.

Supplementary Note 2. Surface properties of BNCs

Similar ligand densities of acetate (2.42 ligand nm^{-2}), polyacrylate (1.92 ligand nm^{-2}), and Mg^{2+} adsorbed polyacrylate (2.57 ligand nm^{-2}) on the BNCs were confirmed by thermogravimetric (TG) analysis (Supplementary Fig. 6, Supplementary Table 1). Two polyacrylate-grafted surfaces had values close to the maximum ligand-to-surface ratio for a coordinated monolayer (2.4–2.7 ligand nm^{-2}), whereas the acetate-grafted surface had a value much lower than the ratio (9 ligands nm^{-2}) needed to fill the BNC surface sufficiently.[16] The Fe^{2+}/Fe^{3+} ratio derived from the Fe $2p_{3/2}$ transition in the Fe $2p$ XPS profile was estimated to be 0.5 even after Mg^{2+} adsorption, maintaining the stoichiometry of Fe_3O_4; this supports that Mg^{2+} mainly exists on the BNC surface rather than replacing the Fe lattice, as also shown in TEM energy-dispersive X-ray spectroscopy (EDS) image (Supplementary Table 3, Supplementary Fig. 8).

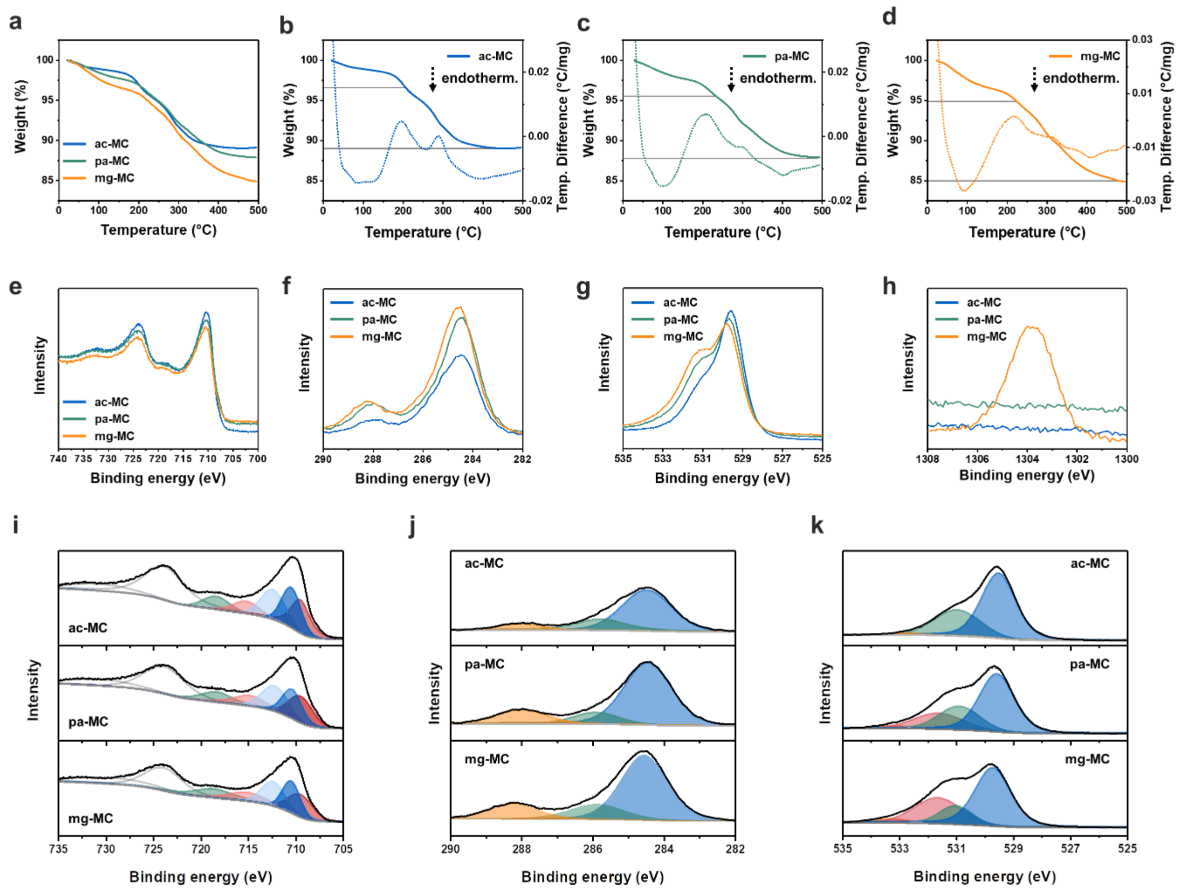

Supplementary Fig. 6 | Surface characterisation of mesocrystals with different surface ligands. a–d, TG-DTA of Fe_3O_4 mesocrystals with different surface ligands: acetate (**b**), polyacrylate (**c**), and Mg^{2+}-adsorbed polyacrylate (**d**). The solid and dotted lines represent the remaining weight percent (TG) and temperature difference (DTA), respectively. The grey solid lines indicate the temperature range for surface ligand decomposition. **e–h**, XPS profiles of Fe_3O_4 mesocrystals at different energy levels: Fe 2*p* (**a**), C 1*s* (**b**), O 1*s* (**c**), and Mg 1*s* (**d**). **i–k** Deconvoluted high-resolution XPS profiles: Fe 2*p* (**i**), with peaks designated as Fe^{3+} satellite peaks (green), Fe^{2+} satellite peaks (pink), Fe^{3+} at tetrahedral sites (sky blue), Fe^{3+} at octahedral sites (blue), and Fe^{2+} at octahedral sites (red) in order of increasing binding energy; C 1*s* (**j**), with peaks marked as COO^- (orange), CO–Fe (green), and sp^3 C (blue) in order of increasing binding energy; and O 1*s* (**k**), with peaks labelled as surface adsorbed H_2O (orange), COO^- (red), Fe–OH surface (green), and Fe–O lattice (blue) in order of increasing binding energy.

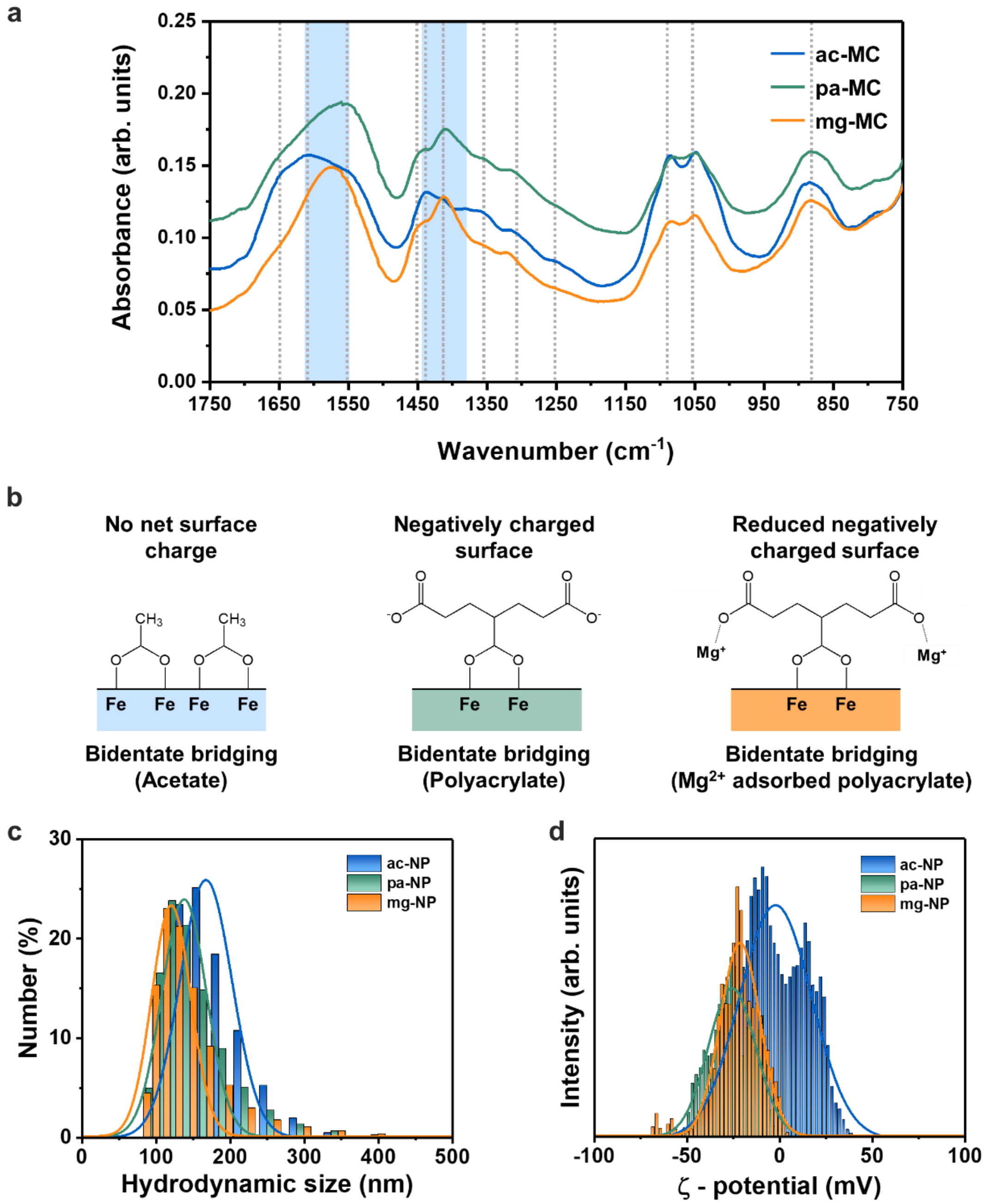

Supplementary Fig. 7 | Coordination of surface ligands on Fe_3O_4 mesocrystals. a, FT-IR spectra of Fe_3O_4 mesocrystals with different surface ligands, demonstrating the bidentate bridging coordination of surface ligands on BNC surfaces. The dotted grey lines indicate the characteristic peaks from acetate and polyacrylate coordinated on the BNC surface, as summarised in Supplementary Table 1. The asymmetric and symmetric stretching vibrations of COO^- groups of acetate and polyacrylate, respectively, are highlighted in blue. **b**, Schematic explaining the conformation of surface ligands on BNC surfaces. **c** and **d**, Hydrodynamic size distribution (**c**) and ζ-potential distribution (**d**) of Fe_3O_4 mesocrystals. The hydrodynamic size of the mesocrystals dispersed in water was measured by DLS, showing that the mesocrystals coordinated by polyacrylate and Mg^{2+}-adsorbed polyacrylate are remarkably dispersed in water without additional surface modification.

Supplementary Table 1. Grafting densities of surface ligands on the BNC surface. wt_{ligand} and wt_{Fe3O4} are the relative mass of ligand and Fe_3O_4 mesocrystal, respectively.

	ac-MC	pa-MC	mg-MC
wt_{ligand} (%)	6.34	7.37	9.02
wt_{Fe3O4} (%)	92.56	90.34	87.47
wt_{ligand}/wt_{Fe3O4}	0.0685	0.0816	0.1031
Grafting density (σ, ligand/nm^2)	2.42	1.92	2.57

Supplementary Table 2. Peak parameters in the FT-IR analysis. v_{as} and v_s indicate asymmetric vibration and symmetric vibration of chemical bonds, respectively.

ac-MC	pa-MC	mg-MC	Peak assignment
1642	1649	1666	Remaining Fe_3O_4 surface (δ(O-H))
1608	-	-	$v_{as}COO^-$ (Fe)
1547	1556	1575	$v_{as}COO^-$
-	1442	1445	CH_2 bending
1439	1439	1439	v_sCOO^- (Fe)
1415	1410	1412	v_sCOO^-
1358	1356	1356	$CH\text{-}CH_3$
1317	1314	1320	C-H deformation
1249	1245	1245	C-H wagging
1108	1112	1112	O-C-C stretching
1083	1083	1086	C-O-C stretching
1049	1050	1049	$C\text{-}CH_3$
1021	1017	1024	C-O stretching
883	883	883	C-C stretching
786	792	788	C-H bending

Supplementary Table 3. Peak parameters in the deconvoluted XPS spectra. ***sp^3* indicates *sp^3* carbons**

Samples	Fe_{2p} binding energy (eV)				O_{1s} binding energy (eV)				C_{1s} binding energy (eV)		
	Fe^{3+} (tetrahedral)	Fe^{3+} (octahedral)	Fe^{2+} (octahedral)	Fe^{2+}/Fe^{3+}	O^{2-}	O^-	COO^-	H_2O	sp^3	CO-Fe	COO^-
ac-MC	712.4	710.4	709.5	0.53	529.5 (67%)	531.0 (32%)	-	532.8 (1%)	284.5 (69%)	285.8 (23%)	288.0 (8%)
pa-MC	712.2	710.4	709.5	0.50	529.6 (56%)	530.9 (24%)	531.6 (19%)	533.3 (2%)	284.4 (72%)	285.9 (12%)	288.0 (16%)
mg-MC	712.4	710.4	709.5	0.50	529.7 (55%)	531.0 (14%)	531.7 (28%)	533.4 (3%)	284.5 (65%)	285.9 (19%)	288.2 (16%)

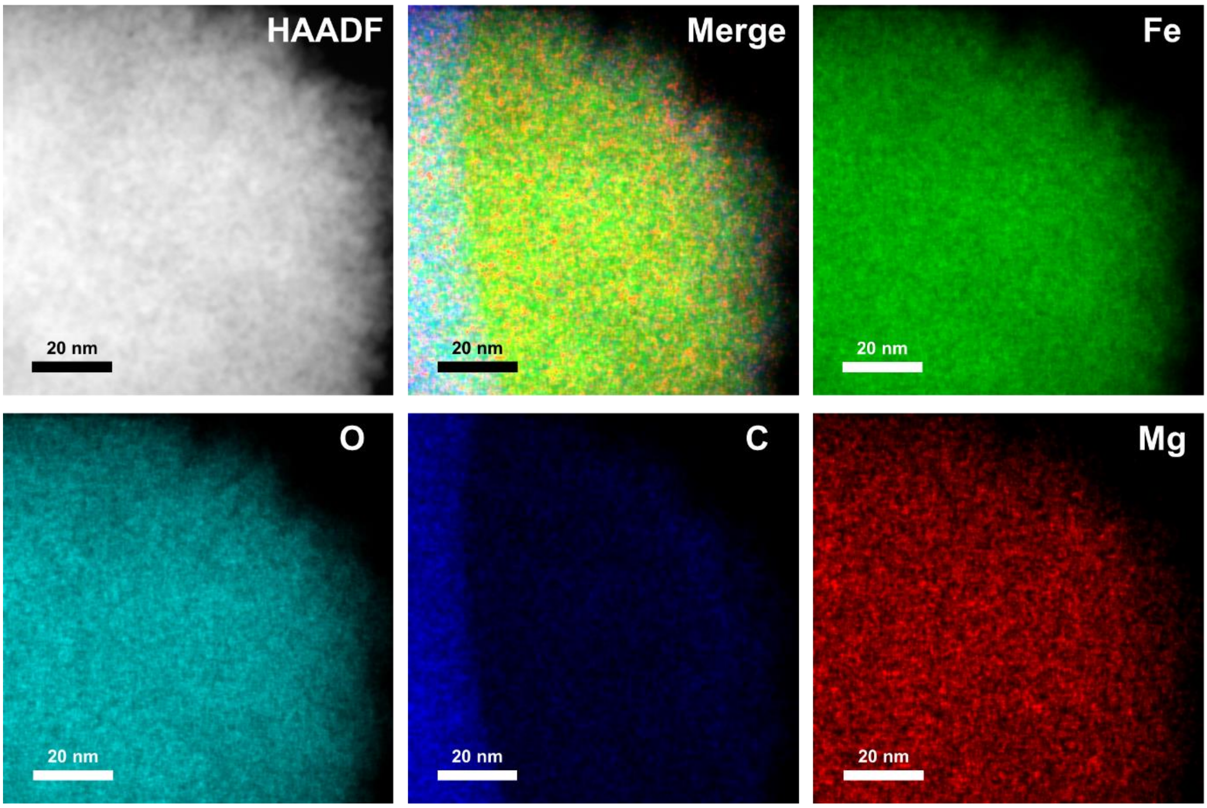

Supplementary Fig. 8 | Elemental mapping of mg-MC. Representative HAADF-STEM and elemental mapping images of mg-MC. The Mg atoms are distributed on the outer surfaces of the Fe_3O_4 mesocrystals. The atomic percentage ratio between Fe and Mg was measured to be 94.98:5.02, which is in good agreement with 93.58:6.42, the combinational result from AAS for the Fe content and ICP-OES for the Mg content.

Supplementary Note 3. Microstructural analysis

To quantify the degree to which the mesocrystals undergo a disorder–order transition according to the type of surface ligands, we introduced the concept of an 'orientation factor'. First, we obtained several SAED patterns from a single mesocrystal without any interference from the surrounding mesocrystal by adjusting the TEM aperture (Supplementary Fig. 9 a,d,g). Then, we converted SAED patterns plotted in the polar coordinate system into a Cartesian coordinate system with a radius (x-axis) and azimuthal angle (y-axis) using Gatan DigitalMicrograph software (Supplementary Fig. 9 b,e,h). Next, we acquired an intensity profile from the spots on a circle with the specific radius indicating the Fe_3O_4 {311} plane, one of the major diffraction patterns in XRD (PDF no. 00-019-0629), as a function of the azimuthal angle ($f(\varphi)$) (Supplementary Fig. 9 c, f, i). From the intensity graph, we finally derived the orientation factor using the equation $\frac{1-\int_0^{\pi} f(\varphi)d\varphi}{\pi}$.

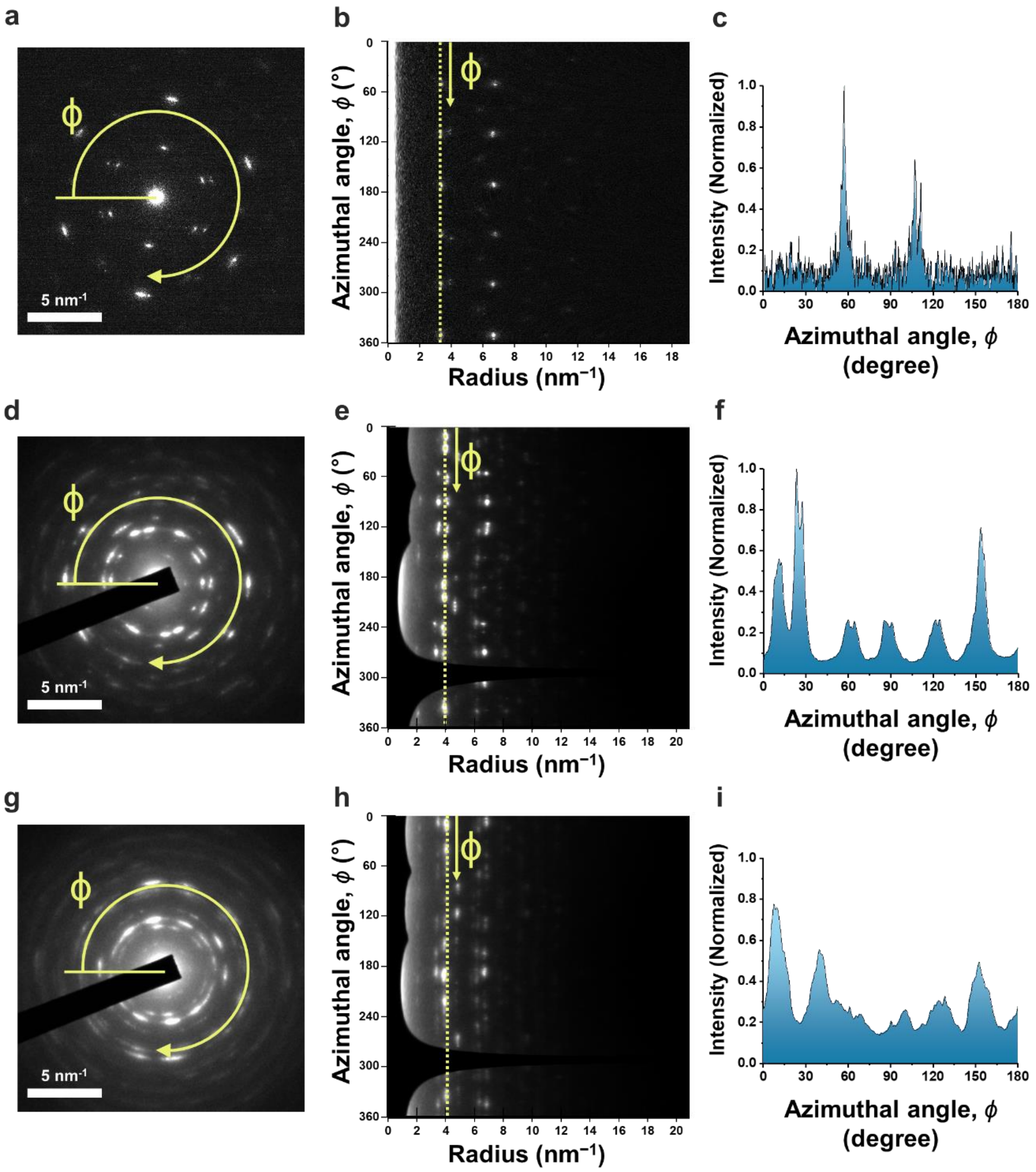

Supplementary Fig. 9 | Derivation of the orientation factor of BNCs constituting a single mesocrystal. **a**, **d**, and **g**, SAED patterns acquired from single mesocrystals with different BNC alignments: ac-MC (**a**), pa-MC (**d**), and mg-MC (**g**). **b**, **e**, and **h**, Diffraction spots as functions of the azimuthal angle (y-axis) and radius in reciprocal space (x-axis). **c**, **f**, and **i**, Orientation factors derived from the azimuthal profiles of the diffraction spots in the {311} plane of Fe_3O_4 in the azimuthal angle range from 0° to 180°.

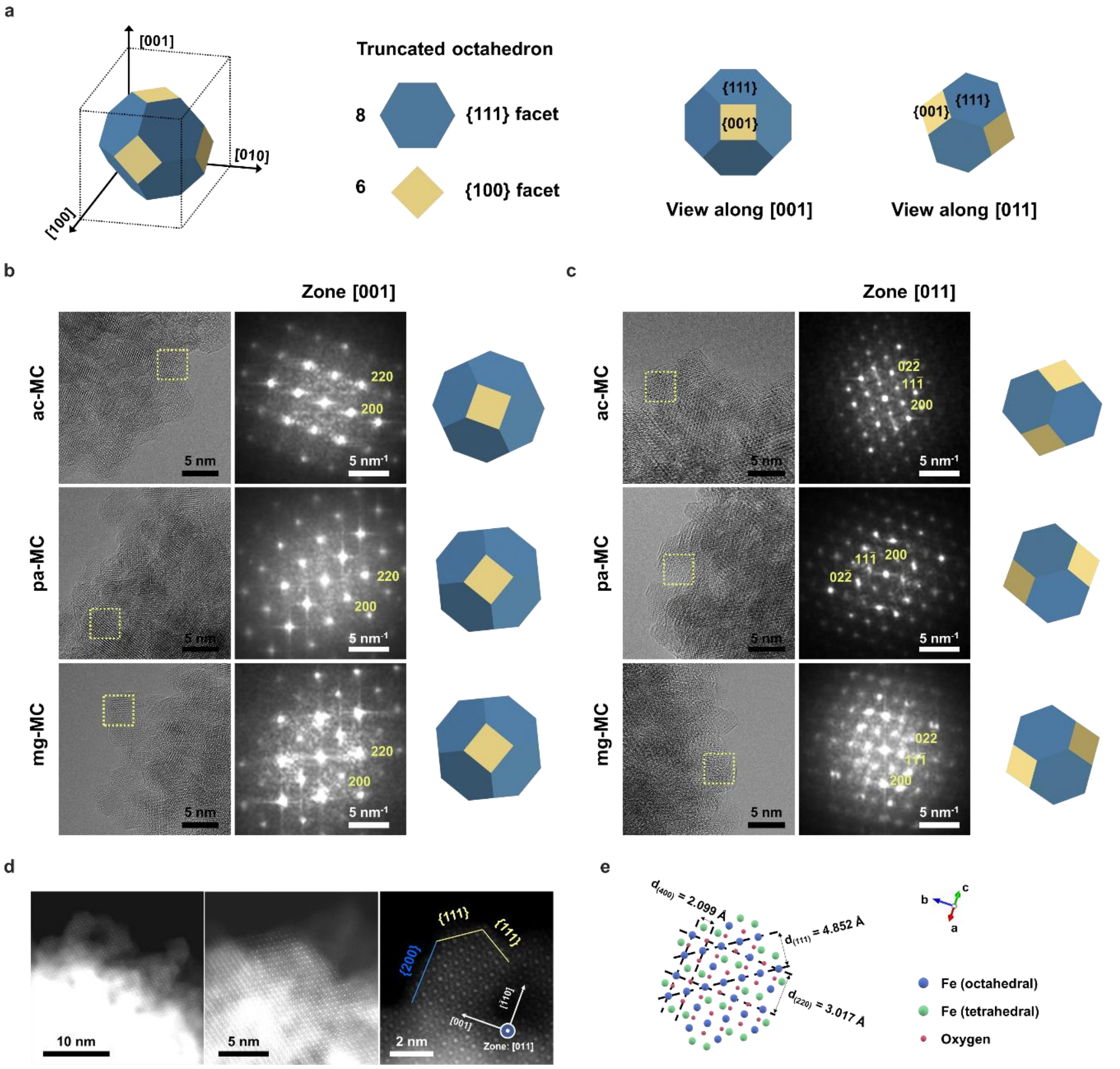

Supplementary Fig. 10 | Morphological and structural analyses of BNCs. a, 3D modelling of a truncated octahedral BNC. **b** and **c**, BNCs from ac-MC (top), pa-MC (middle), and mg-MC (bottom) with different zone axes: [001] zone axis (**b**), and [011] zone axis (**c**). The FFT patterns were acquired from the yellow dotted box. The final BNC structure was derived from the faceted hexagon and octagon corresponding to the [001] and [011] projections of the truncated octahedron, respectively. **d**, HAADF-STEM image of BNCs. The yellow and blue lines indicate the faceted edges of the BNC with the {200} and {111} planes, respectively. **e**, Atomic configuration of the Fe_3O_4 BNC at the [011] zone axis.

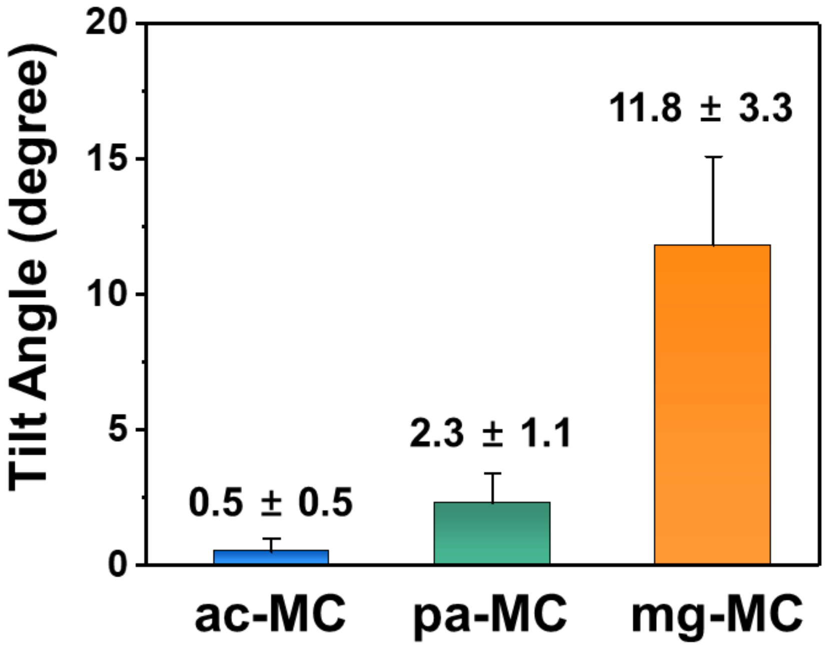

Supplementary Fig. 11 | Statistical tilt angles between adjacent BNCs in each sample. The tilt angle between adjacent BNCs was measured using the same method in Fig. 5. The data bars are the average values from n = 6, excluding the maximum and minimum values. The error bars indicate the standard deviations.

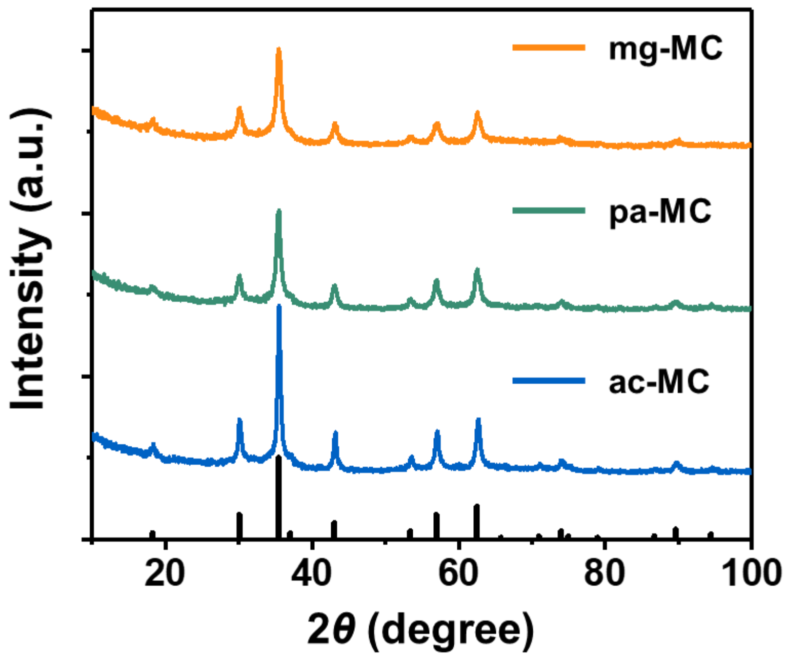

Supplementary Fig. 12 | Microstructural analysis of Fe_3O_4 mesocrystals with different BNC alignments. XRD patterns of Fe_3O_4 mesocrystals corresponding to the reference powder XRD patterns of magnetite (PDF no. 00-019-0629). The crystallite sizes calculated from the diffraction peak of the (311) plane at 35.4° using the Debye–Scherrer equation are 15.5 nm (ac-MC), 9.8 nm (pa-MC), and 8.4 nm (mg-MC).

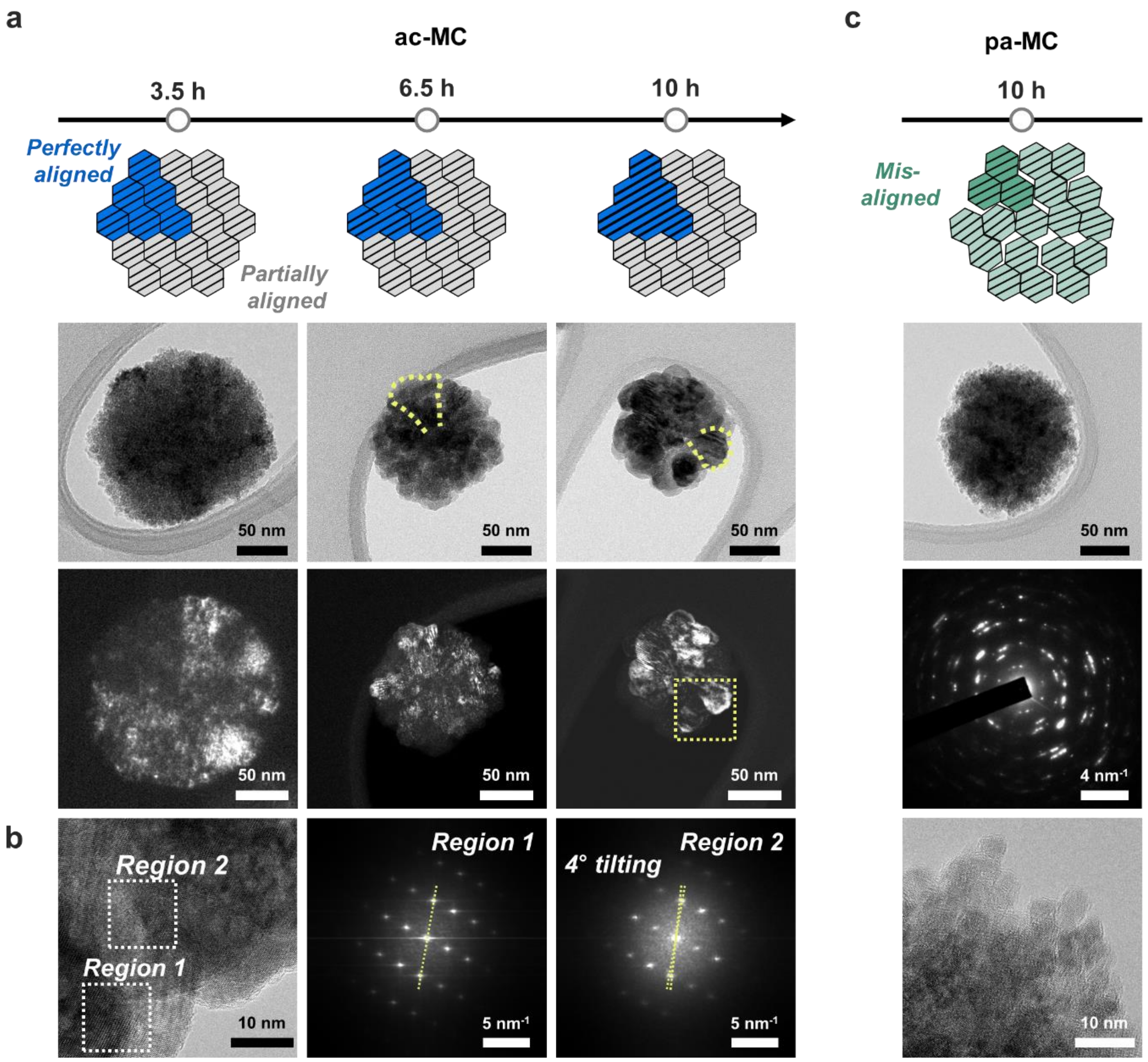

Supplementary Fig. 13 | Development of multiple grains via the coarsening of the aligned BNCs within the mesocrystals during postreaction. a, Schematics and TEM images of ac-MC at reaction times of 3.5, 6.5, and 10 h. The yellow dotted line in the BF-TEM image indicates a region with perfectly aligned BNCs, as shown in the corresponding DF images below. The DF-TEM images of ac-MC constructed from a single diffraction spot show that the perfectly aligned and partially aligned regions are clearly distinguished in a single mesocrystal. Interestingly, the crystallographically identical region maintains its size during the postreaction, but the BNCs in this region are coarsened, producing larger grains. **b**, HR-TEM image and FFT patterns of coarsened grains in the area enclosed by the yellow dotted box in the DF image at 10 h. The FFT patterns acquired from Regions 1 and 2 represent 4° tilting, indicating that the region with perfectly aligned BNCs within a mesocrystal is distinguished by partial misalignment with its neighbour. **c**, Schematic, TEM images, and SAED pattern of pa-MC 10 h postreaction. The misaligned BNCs are not coarsened and maintain their discernible nanogranular morphology.

Supplementary Note 4. Magnetic property analysis

Supplementary Table 4. Magnetic properties of Fe_3O_4 mesocrystals.

	ac-MC	pa-MC	mg-MC
M_s (Am^2/kg)	87.7	82.0	74.4
H_c (kA/m)	1.88	0.77	0.47
M_r (Am^2/kg)	2.83	1.29	0.74
T_B (K)	> 400	325.3	215.6

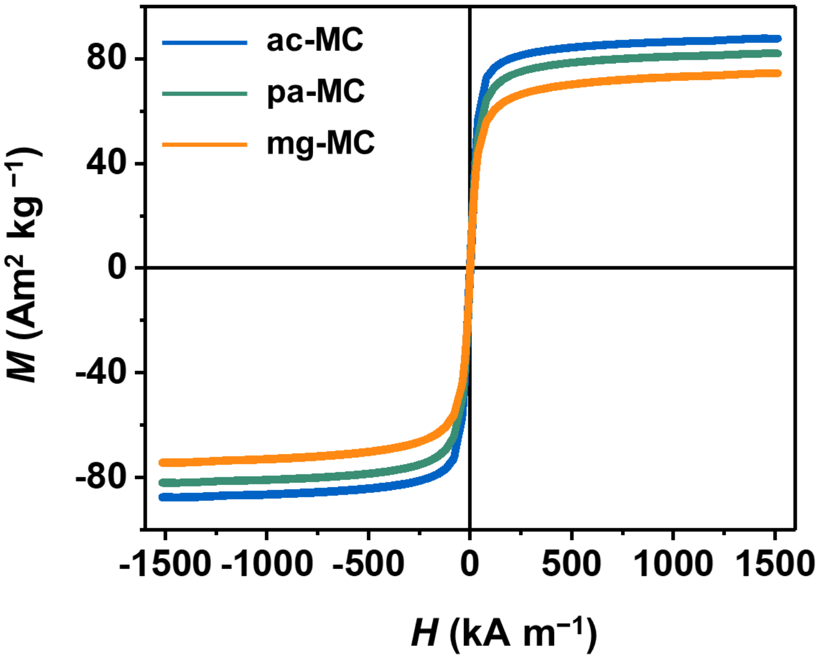

Supplementary Fig. 14 | Magnetic hysteresis curves of Fe_3O_4 mesocrystals at room temperature.

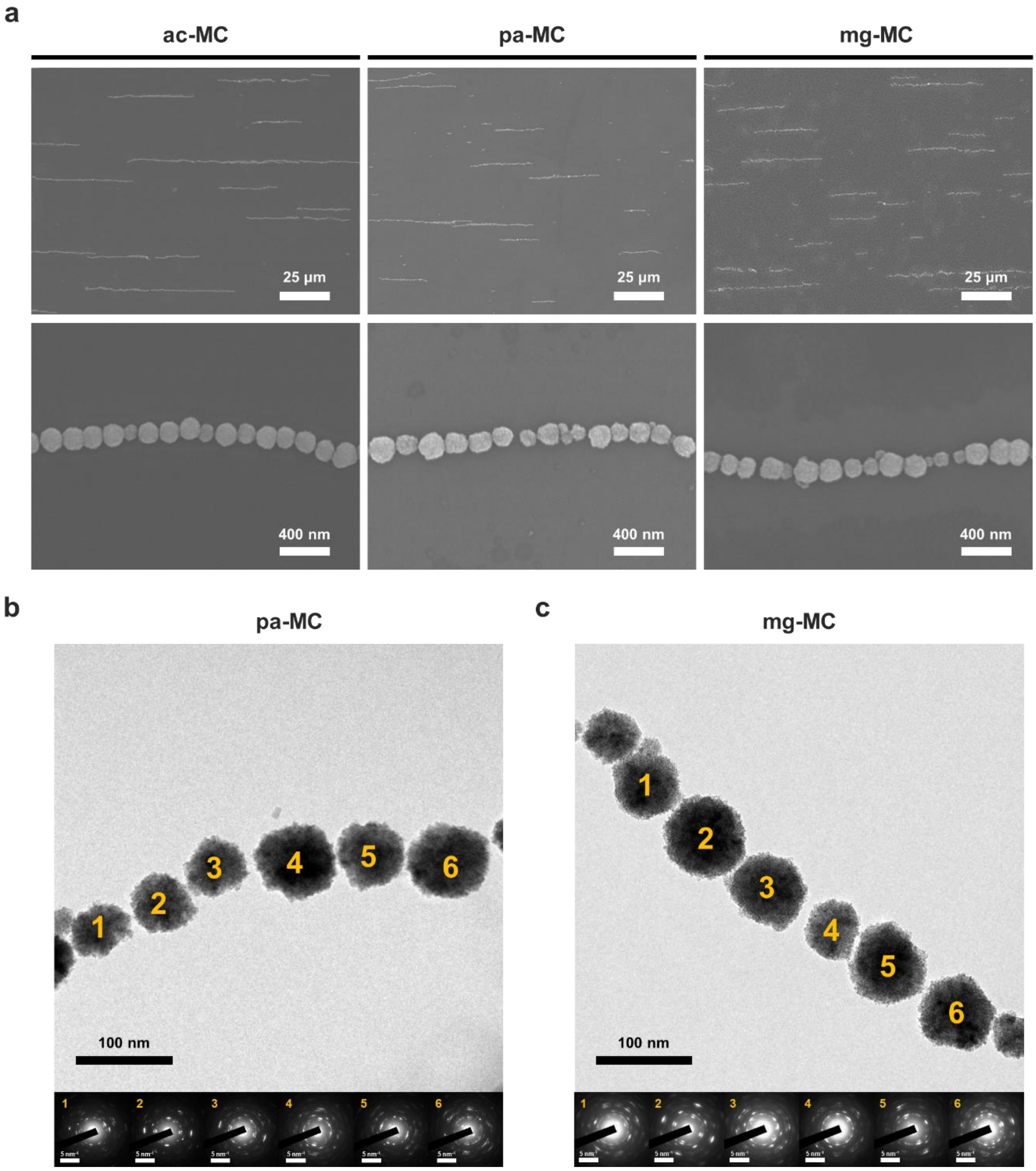

Supplementary Fig. 15 | Formation of 1-D Fe_3O_4 nanochains. a, SEM images of 1-D nanochains consisting of mesocrystals with different crystallographic orders. The nanochains were sufficiently separated from each other by controlling the concentration of Fe_3O_4 mesocrystals to minimise magnetostatic interactions. **b** and **c**, TEM images of 1-D chains illustrating the crystallographic orientations of individual mesocrystals with different BNC alignments: pa-MC (**b**), and mg-MC (**c**). The corresponding SAED patterns were acquired from the particles marked with the same numbers by carefully positioning the aperture to avoid any interference from the adjacent mesocrystals. Compared with the orientational arrangement in the 1-D chain of the ac-MCs, the degree of arrangement of the pa-MCs and mg-MCs along the axial direction of the 1-D chain gradually decreases as the interaction between BNCs decreases.

Supplementary Note 5. Driving force analysis

1. Inter-BNC interaction potential analysis using the Derjaguin–Landau–Verwey–Overbeek (DLVO) model.

We examined the pair interaction potential between two BNCs using the DLVO theory as follows:

$$U = U_{\mathrm{vdW}} + U_{\mathrm{elec}} + U_{\mathrm{mag}},$$

where U_{VdW}, U_{elec}, and U_{mag} represent the potentials contributed by van der Waals, electrostatic, and magnetic dipole–dipole interactions, respectively. The van der Waals interactions between BNCs were calculated using the following general equation:

$$U_{\mathrm{vdW}} = -\frac{A}{6}\left[\frac{2r^2}{(4r+D)D} + \frac{2r^2}{(2r+D)^2} + ln\frac{(4r+D)D}{(2r+D)^2}\right],$$

where A is the Hamaker constant, r is the BNC radius, and D is the plane-to-plane distance between BNCs. We calculated the nonretarded Hamaker constant based on the Lifshitz theory.[17] For identical BNCs (labelled 1) interacting across medium 3, the equation for the nonretarded Hamaker constant is as follows:

$$\mathrm{A} = \frac{3}{4}k_B T\left(\frac{\varepsilon_1-\varepsilon_3}{\varepsilon_1+\varepsilon_3}\right)^2 + \frac{3hv_e}{16\sqrt{2}}\frac{(n_1{}^2-n_3{}^2)^2}{(n_1{}^2+n_3{}^2)^{3/2}},$$

where k_B is the Boltzmann constant, ε_i is the dielectric constant of Fe_3O_4 (20,000) and the medium (42.6 for ethylene glycol–water mixture), h is the Planck constant, υ_e is the plasma frequency of Fe_3O_4 (4.0×10^{15} Hz), and n_i is the refractive index of Fe_3O_4 (1.97) and the medium (1.43).[18–21] The Hamaker constant for our system was calculated to be 8.55×10^{-20} J.

The contribution of the electrostatic interaction depending on the type of surface ligand was obtained from the following equation, which is a simplified form valid for a two-sphere model at a low surface potential of approximately 25 mV:

$$U_{\mathrm{elec}} = 2\pi r\varepsilon_0\varepsilon_r\psi_0{}^2 e^{-\kappa D},$$

where the surface potential Ψ_0 is assumed to be the ζ-potential of BNCs coordinated with different surface ligands; ε_0 and ε_r are the vacuum permittivity (8.85×10^{-12}) and relative permittivity (42.6) of the solution, respectively; and κ is the inverse Debye length.[17] The radii r of the BNCs of the ac-MCs, pa-MCs, and mg-MCs were measured from TEM images to be 1.99, 1.67, and 1.71 nm, respectively.

The Debye length, κ^{-1}, is given by

$$\kappa^{-1} = (\sum_i \frac{\rho_\infty e^2 z_i^2}{\varepsilon_0 \varepsilon_r k_B T})^{-1/2} = (\sum_i \frac{1000 N_A e^2 M_i z_i^2}{\varepsilon_0 \varepsilon_r k_B T})^{-1/2},$$

$$\kappa^{-1} = \left[\frac{1000 N_A e^2([I]_{Na} z_{Na}^2 + [I]_{Cl} z_{Cl}^2)}{\varepsilon_0 \varepsilon_r k_B T}\right]^{-1/2} = \left[\frac{1000 N_A e^2 (0.1138+0.1138)}{\varepsilon_0 \varepsilon_r k_B T}\right]^{-1/2} = 0.66 \text{ nm}$$

for the BNCs coordinated by acetate,

$$\kappa^{-1} = \left[\frac{1000 N_A e^2([I]_{Na} z_{Na}^2 + [I]_{Cl} z_{Cl}^2)}{\varepsilon_0 \varepsilon_r k_B T}\right]^{-1/2} = \left[\frac{1000 N_A e^2 (0.1233+0.1138)}{\varepsilon_0 \varepsilon_r k_B T}\right]^{-1/2} = 0.65 \text{ nm}$$

for the BNCs coordinated by polyacrylate, and

$$\kappa^{-1} = \left[\frac{1000 N_A e^2([I]_{Na} z_{Na}^2 + [I]_{Cl} z_{Cl}^2 + [I]_{Mg} z_{Mg}^2)}{\varepsilon_0 \varepsilon_r k_B T}\right]^{-1/2} = \left[\frac{1000 N_A e^2 (0.1233+0.1518+0.0190)}{\varepsilon_0 \varepsilon_r k_B T}\right]^{-1/2} = 0.54 \text{ nm}$$

for the BNCs coordinated by Mg^{2+}-adsorbed polyacrylate, where ρ is the number density, e is the electron charge, and $[I]_i$ and z_i are the molar concentration and valency of ions, respectively. The Debye screening length formed by multi-ionic electrolytes can screen the charge–charge interactions at separation distances D larger than 0.66, 0.65, and 0.54 nm for ac-MCs, pa-MCs, and mg-MCs, respectively.

Given that Fe_3O_4 mesocrystals exhibit superparamagnetic properties when they are smaller than 10 nm, we should consider the dipole–dipole interactions between BNCs with sizes of approximately 4 nm, which is known as the Keesom interaction and is described by

$$U_{mag} = -\frac{1}{3k_B T}\left[\frac{(\mu_0 V M)^2}{4\pi\mu_0 r}\right]^2,$$

where μ_0 is the vacuum permeability constant (1.26×10^{-12}), V is the BNC volume, M is the saturation magnetisation of the BNC (measured by vibrating sample magnetometry: $M = 4.5 \times 10^5$, 4.2×10^5, and 3.9×10^5 A m^{-1} for ac-MC, pa-MC, and mg-MC, respectively). Because the BNCs are ultrasmall, $U_{vdW} \gg U_{mag}$.[22]

2. Surface energy and binding affinity of surface ligands calculated via DFT

Computational details. Density functional theory (DFT) calculations were performed using the generalised gradient approximation with Perdew–Burke–Ernzerhof parameterisation and Quantum Espresso from Materials Square (a Web-based DFT calculation platform). We performed bulk calculations using the rhombohedral primitive unit cell (14 atoms). We utilised 8 × 8 × 8 k-point grids and a 90 Ry energy cut-off for the wave function, which ensured electronic and ionic convergence. The convergence criteria of the structural relaxation and

electronic self-consistency for energy and forces set were chosen to be 10^{-8} Ry and 0.000038 Ry Bohr^{-1}, respectively. In this setup, the lattice constant a of magnetite was calculated to be 0.8483 nm, in close agreement with the experimental value of 0.8396 nm.[23,24] The calculated lengths of the two kinds of Fe–O bonds (Fe_{tet}–O and Fe_{oct}–O) were 0.193 nm and 0.207 nm, in good agreement with experimental values of 0.188 nm and 0.207 nm, respectively.[23] The calculated total spin magnetisation per formula unit was 3.72 μ_B, in reasonable agreement with the experimental value of 4.05 μ_B.[23,24] For the slab calculations, the Fe_3O_4 (001), (110), and (111) surfaces were modelled as a 1 × 1 × 2 expanded supercell (112 atoms). A 20 Å-thick vacuum layer was placed within the periodic cells repeated along the z-axis to obviate interactions. We used a 2 × 2 × 1 k-point mesh and 90 Ry energy cut-off of the wave function from the unit cell (112 atoms). We considered van der Waals interactions using the DFT-D3(BJ) method. All calculations were spin-polarised. The convergence criteria of the structural relaxation and electronic self-consistency for energy and forces set were chosen to be 10^{-7} Ry and 0.00038 Ry Bohr^{-1}, respectively.

Surface energy calculation. The surface structures of Fe_3O_4 were previously reported to be (001), (110), and (111).[23] This study showed that six surfaces have (111) terminations, two have (110) terminations, and two others have (001) terminations. Another investigation revealed that the surface energy follows the order (001) < (111) < (110) according to DFT calculations.[24] Therefore, the present surface models were selected as the termination with the lowest surface energy of individual surface indices.

The surface energy, E_{surface}, was calculated as follows:

$$E_{\mathrm{surface}} = (E_{\mathrm{slab}} - E_{\mathrm{bulk}})/2A,$$

where E_{slab} is the total energy of the slab, E_{bulk} is the total energy of the bulk, and A is the surface area of each side of the slab.

When structural relaxation was performed, all slab models were fully relaxed without the fixed layer. After structural relaxation, the (001), (110), and (111) surface energies were calculated to be 0.51, 4.24, and 1.12 eV nm^{-2}, respectively (Table 1).

Binding energy calculation for organic molecules. A significant factor affecting the OA of mesocrystals is surface anisotropy. To clarify the effects of the surface anisotropy on the OA of Fe_3O_4 mesocrystals in a particular environment, we calculated the binding energies of acetate, polyacrylate (n = 3), and polyacrylate with Mg cations on the Fe_3O_4 (001), (110), and (111)

surfaces, respectively. For the polyacrylate configuration, we experimentally determined that one carboxylate anion was absorbed with bidentate bridging between Fe atoms on the Fe_3O_4 surface and that the other carboxylate anion was not bonded in the aqueous solution. For the DFT calculations, we assumed that the acrylate molecule had three carboxylate anions owing to size limitations.

The binding energies of acetate, polyacrylate, and polyacrylate with Mg cations on the Fe_3O_4 (001), (110), and (111) surfaces were calculated as follows:

$E_{\text{binding}}^{\text{acetate}} = E_{\text{total}} - E_{\text{acetate}} - E_{\text{surface}}$,

$E_{\text{binding}}^{\text{acrylate}} = E_{\text{total}} - E_{\text{acrylate}} - E_{\text{surface}}$,

and $E_{\text{binding}}^{\text{acrylate with Mg}} = E_{\text{total}} - E_{\text{acrylate}} - E_{\text{Mg}} - E_{\text{surface}}$,

where E_{surface}, E_{acetate}, E_{acrylate}, E_{Mg}, and E_{total} are the energies of the surface, acetate, acrylate, Mg atom, and all molecules absorbed on the surface, respectively. Each individual energy term on the right side can be obtained directly from DFT calculations. Table 1 presents the calculation results.

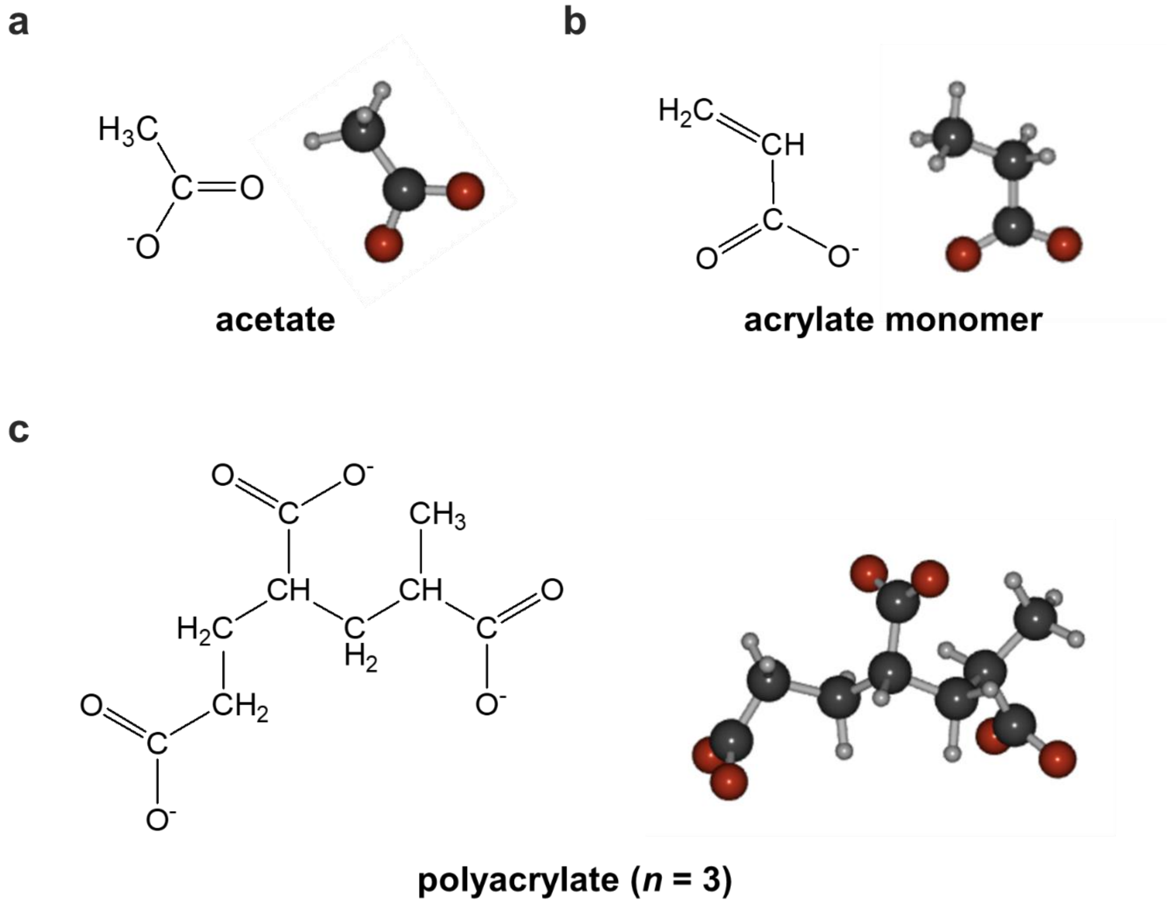

Supplementary Fig. 16 | Surface ligand models for DFT calculations. a, Acetate. **b**, Acrylate monomer, **c**, polyacrylate (n = 3) molecule for a unit model of polyacrylate. The black, white, and red spheres represent C, H, and O atoms, respectively.

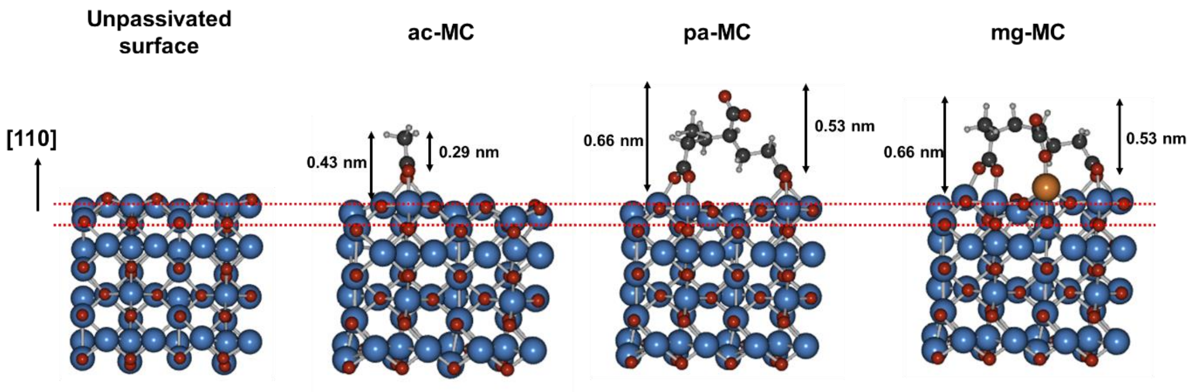

Supplementary Fig. 17 | Surface ligand configuration on the (110) surface of the BNC. Side view of a BNC slab simulated by DFT calculations. The blue, black, white, red, and orange spheres indicate Fe, C, H, O, and Mg atoms, respectively. The red dotted lines are visual guides illustrating the surface distortion relative to the unpassivated Fe_3O_4 surface.

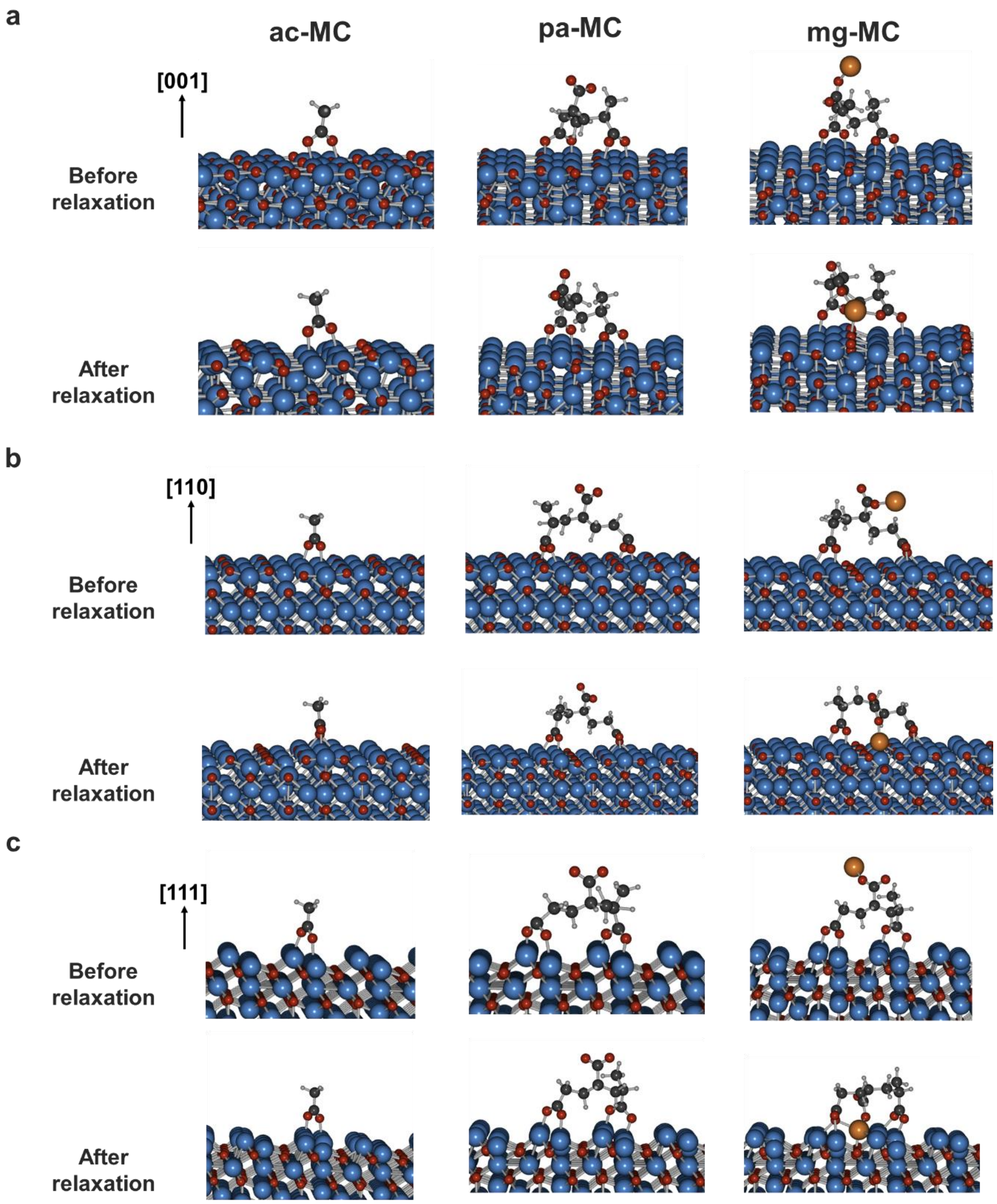

Supplementary Fig. 18 | Simulated configurations of organic molecules on the Fe_3O_4 BNC. a–c, Side views of Fe_3O_4–organic ligand configuration at the (100) (**a**), (110) (**b**), and (111) (**c**) planes of Fe_3O_4 with different surface conditions: ac-MC (left), pa-MC (middle), and mg-MC (right). The blue, black, white, red, and orange spheres represent Fe, C, H, O, and Mg atoms, respectively.

Supplementary References

1. Park, B. C. *et al.* Strategy to control magnetic coercivity by elucidating crystallization pathway-dependent microstructural evolution of magnetite mesocrystals. *Nat. Commun.* **11**, 298 (2020).
2. Oswald, W. Studien uber die Bildung und Umwandlung fester Korper. *Z. Phys. Chem* **22**, 289-330 (1897).
3. Vali, H. *et al.* Formation of tabular single-domain magnetite induced by Geobacter metallireducens GS-15. *Proc. Natl. Acad. Sci.* **101**, 16121-16126 (2004).
4. Hansel, C. M., Benner, S. G. & Fendorf, S. Competing Fe(II)-induced mineralization pathways of ferrihydrite. *Environ. Sci. Technol.* **39**, 7147-7153 (2005).
5. Ahn, T., Kim, J. H., Yang, H.-M., Lee, J. W. & Kim, J.-D. Formation pathways of magnetite nanoparticles by coprecipitation method. *J. Phys. Chem. C* **116**, 6069-6076 (2012).
6. Baumgartner, J. *et al.* Nucleation and growth of magnetite from solution. *Nat. Mater.* **12**, 310-314 (2013).
7. Mirabello, G. *et al.* Crystallization by particle attachment is a colloidal assembly process. *Nat. Mater.* **19**, 391-396 (2020).
8. Zhu, G. *et al.* Self-similar mesocrystals form via interface-driven nucleation and assembly. *Nature* **590**, 416-422 (2021).
9. Schwertmann, U. Solubility and dissolution of iron oxides. *Plant Soil* **130**, 1-25 (1991).
10. Stefánsson, A. Iron(III) hydrolysis and solubility at 25 °C. *Environ. Sci. Technol.* **41**, 6117-6123 (2007).
11. Navrotsky, A., Mazeina, L. & Majzlan, J. Size-driven structural and thermodynamic complexity in iron oxides. *Science* **319**, 1635-1638 (2008).
12. Navrotsky, A. Energetic clues to pathways to biomineralization: Precursors, clusters, and nanoparticles. *Proc. Natl. Acad. Sci.* **101**, 12096-12101 (2004).
13. Avrami, M. Kinetics of phase change. II transformation-time relations for random distribution of nuclei. *J. Chem. Phys.* **8**, 212-224 (1940).
14. Jeon, K.-J. *et al.* Air-stable magnesium nanocomposites provide rapid and high-capacity hydrogen storage without using heavy-metal catalysts. *Nat. Mater.* **10**, 286-290 (2011).
15. Xiang, K., Yang, K., Carter, W. C., Tang, M. & Chiang, Y.-M. Mesoscopic phase transition kinetics in secondary particles of electrode-active materials in lithium-ion batteries. *Chem. Mater.* **30**, 4216-4225 (2018).

16. Zhang, X. *et al.* Conformation-dependent coordination of carboxylic acids with Fe_3O_4 nanoparticles studied by ATR-FTIR spectral deconvolution. *Langmuir* **35**, 5770-5778 (2018).
17. Israelachvili, J. N. *Intermolecular and Surface Forces* (Academic Press, Waltham, MA, 2011).
18. Sverjensky, D. A. & Sahai, N. Theoretical prediction of single-site enthalpies of surface protonation for oxides and silicates in water. *Geochim. Cosmochim. Acta* **62**, 3703-3716 (1998).
19. Akerlof, G. Dielectric constants of some organic solvent-water mixtures at various temperatures. *J. Am. Chem. Soc.* **54**, 4125-4139 (1932).
20. Faure, B., Salazar-Alvarez, G. & Bergstrom, L. Hamaker constants of iron oxide nanoparticles. *Langmuir* **27**, 8659-8664 (2011).
21. Fogg, E. T., Hixson, A. N. & Thompson, A. R. Densities and refractive indexes for ethylene glycol-water solutions. *Anal. Chem.* **27**, 1609-1611 (1955).
22. Wu, L. *et al.* High-temperature crystallization of nanocrystals into three-dimensional superlattices. *Nature* **548**, 197-201 (2017).
23. Tao, Y. *et al.* Surface structures of Fe_3O_4 (111), (110), and (001): A density functional theory study. *J. Fuel Chem. Technol.* **38**, 121-128 (2010).
24. Santos-Carballal, D., Roldan, A., Grau-Crespo, R. & de Leeuw, N. H. A DFT study of the structures, stabilities and redox behaviour of the major surfaces of magnetite Fe_3O_4. *Phys. Chem. Chem. Phys.* **16**, 21082-21097 (2014).
